# Aptamer and DNAzyme Based Colorimetric Biosensors for Pathogen Detection

**DOI:** 10.1002/anie.202418725

**Published:** 2024-11-25

**Authors:** Rudi Liu, Jiuxing Li, Bruno J. Salena, Yingfu Li

**Affiliations:** ^1^ Department of Biochemistry and Biomedical Sciences Michael G. DeGroote Institute of Infectious Disease Research School of Biomedical Engineering Biointerfaces Institute McMaster University 1280 Main Street West Hamilton Ontario L8S 4K1 Canada; ^2^ Department of Medicine McMaster University 1280 Main Street West Hamilton Ontario L8S 4K1 Canada

**Keywords:** Aptamer, DNAzyme, Colorimetric Biosensors, Bacteria, Viruses

## Abstract

The detection of pathogens is critical for preventing and controlling health hazards across clinical, environmental, and food safety sectors. Functional nucleic acids (FNAs), such as aptamers and DNAzymes, have emerged as versatile molecular tools for pathogen detection due to their high specificity and affinity. This review focuses on the in vitro selection of FNAs for pathogens, with emphasis on the selection of aptamers for specific biomarkers and intact pathogens, including bacteria and viruses. Additionally, the selection of DNAzymes for bacterial detection is discussed. The integration of these FNAs into colorimetric biosensors has enabled the development of simple, cost‐effective diagnostic platforms. Both non‐catalytic and catalytic colorimetric biosensors are explored, including those based on gold nanoparticles, polydiacetylenes, protein enzymes, G‐quadruplexes, and nanozymes. These biosensors offer visible detection through color changes, making them ideal for point‐of‐care diagnostics. The review concludes by highlighting current challenges and future perspectives for advancing FNA‐based colorimetric biosensing technologies for pathogen detection.

## Graphical abstract

Pathogen detection is crucial for preventing and managing health risks. This review consolidates the selection of functional nucleic acids (FNAs) for pathogen detection and the development of cost‐effective, simple diagnostic applications. It focuses on integrating FNAs with gold nanoparticles, polydiacetylenes, protein enzymes, G‐quadruplexes, and nanozymes in colorimetric biosensors.

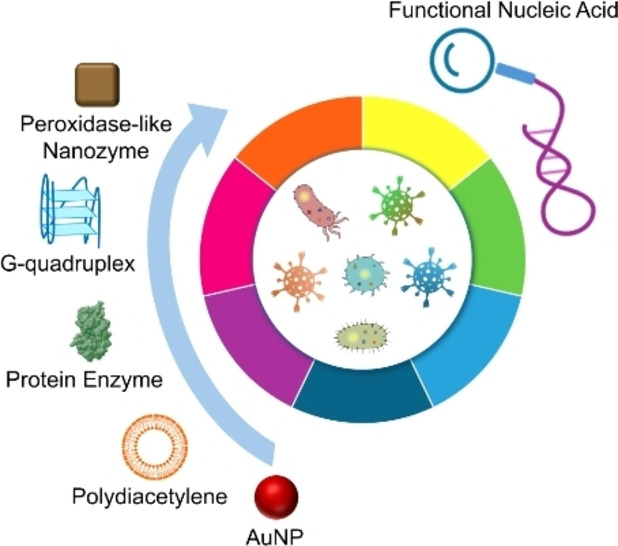

## 1 Introduction

The emergence of the coronavirus disease 2019 (COVID‐19) pandemic, driven by severe acute respiratory syndrome coronavirus 2 (SARS‐CoV‐2), has reignited an unprecedented surge in research on communicable diseases caused by pathogens.^[1]^ Over the past few decades, we have witnessed several severe infectious viral outbreaks, including the severe acute respiratory syndrome coronavirus (SARS‐CoV) in 2003,^[2]^ swine flu in 2009,^[3]^ Middle East respiratory syndrome coronavirus (MERS‐CoV) in 2012,^[4]^ the Ebola virus in West Africa in 2013,^[5]^ and the Zika virus in 2015.^[6]^ Similarly, bacterial pathogens remain a growing global burden, with substantial impacts on both public health and the economy.^[7]^ Notable examples include *Escherichia coli* (*E. coli*), *Clostridium difficile* (*C. difficile*), *Salmonella typhimurium* (*S. typhimurium*), *Listeria monocytogenes* (*L. monocytogenes*), *Mycobacterium tuberculosis* (*M. tuberculosis*), and *Staphylococcus aureus* (*S. aureus*). The diseases caused by these bacteria include diarrhea, tuberculosis, cholera, botulism, and pneumonia.^[8]^ In the United States alone, it is reported that at least 2.8 million people are infected with antibiotic‐resistant bacteria annually, resulting in over 35,000 deaths.^[9]^ Pathogenic diseases accounted for approximately 14 % of total disease‐related mortality worldwide in 2023.^[10]^ The severity and high mortality associated with bacterial and viral pathogens pose significant challenges to public health and the global economy. A critical factor in controlling the large‐scale transmission of these communicable diseases and in tailoring treatments for infected patients is the development of rapid, sensitive, and specific methods for pathogen detection.

Conventional pathogen detection methods are generally classified into three main categories: cell culturing, immunoassays, and nucleic acid amplification.[^11^, ^12^] Among these, cell culturing is considered the most reliable method for bacterial detection.^[13]^ This approach involves growing viable organisms, followed by differentiation tests to identify bacterial species or strains. Although regarded as the gold standard, the long turnaround time, the need for specialized expertise, and the complexity of the procedure make it impractical for routine clinical use for the detection of diverse pathogens.^[14]^ Immunoassays, such as enzyme‐linked immunosorbent assay (ELISA), are simple and rapid, requiring no specialized facilities, unlike bacterial culture.^[15]^ Immunoassays are antibody‐based techniques that detect pathogens by recognizing specific antigens or antibodies produced by the host. A representative example is the lateral flow test strip that has been widely employed for the detection of SARS‐CoV‐2.^[16]^ However, immunoassays cannot differentiate between live and dead pathogens and are prone to false‐negative results due to limited detection sensitivity.[^17^, ^18^] Nucleic acid amplification approaches, including polymerase chain reaction (PCR) and loop‐mediated isothermal amplification (LAMP), are widely used in clinical settings due to their high sensitivity and excellent specificity.^[15]^ However, these methods require complex sample preparation, well‐trained personnel, and sophisticated instrumentation, limiting their utility in resource‐limited settings. Given these challenges, there is an increasing demand for affordable, rapid, sensitive, and specific methods for identifying new or reemerging infectious pathogens.

In recent years, biosensors have gained significant attention as powerful emerging analytical tools, with broad applications in healthcare,^[19]^ the food industry,^[20]^ and environmental monitoring.[^21^, ^22^] The development of easy‐to‐use, rapid, sensitive, and reliable biosensors has addressed challenges associated with traditional pathogen detection. Typically, a biosensor integrates a molecular recognition element (MRE) specific to the analyte with a transducer component, which converts the biomolecular interaction into a measurable physical signal, such as colorimetric, fluorescent, surface‐enhanced Raman spectroscopy, or electrochemical signals.^[23]^ The detection of biological analytes, including DNA/RNA, proteins, viruses, and bacteria, depends largely on the specific binding of MREs with the targets. Functional nucleic acids (FNAs) are a diverse group of RNA and DNA molecules that perform specific functions beyond merely carrying genetic information. To date, many man‐made FNAs, including aptamers, ribozymes and DNAzymes, have emerged as attractive MREs in biosensors for analyzing a wide range of targets, including ions, proteins, viruses and bacteria.[^24^, ^25^, ^26^, ^27^] These short nucleic acid molecules (20–80 nucleotides) are usually derived for a specific function, such as recognizing a specific molecule or catalyzing a chemical reaction, through systematic evolution of ligands by exponential enrichment (SELEX) from a synthetic nucleic acid library.[^27^, ^28^] FNA‐based colorimetric biosensors offer significant potential for advancing point‐of‐care diagnostics, as they enable the rapid detection of harmful pathogens, facilitating timely intervention and mitigating the risk of potential pandemic outbreaks.[^29^, ^30^] Herein, we will summarize the methods for isolating aptamers and DNAzymes specific to pathogens, with a focus on novel selection strategies aimed at deriving FNAs with high sensitivity and specificity. Following this, we will review recent advancements in FNA‐based colorimetric biosensors for pathogen detection.

## 2 In vitro selection of FNAs for pathogens

Until the early 1980s, RNA molecules were considered solely responsible for storing hereditary material and transmitting genetic information within living cells. However, the ground‐breaking discovery by Thomas Cech^[31]^ and Sidney Altman^[32]^ revealed that RNA also possesses catalytic properties. These natural RNA enzymes, termed ribozymes, play both informational and catalytic roles in biological processes. About 20 years later, another type of naturally occurring FNAs with regulatory functions, namely riboswitches, was discovered.^[33]^ Riboswitches can recognize cellular metabolites and undergo an allosteric switch upon binding to them. This structural transformation consequently either promotes or inhibits transcription or expression of the encoded protein, thereby influencing the cellular processes.[^34^, ^35^]

In addition to the discovery of these natural FNAs, the scientific community has devoted significant efforts towards developing artificial FNAs to broaden in vivo or vitro applications. Generally, artificial FNAs comprise DNA or RNA aptamers with ligand‐binding capability, as well as ribozymes and deoxyribozymes (DNAzymes) with catalytic functions (which are collectively termed nucleic acid enzymes). In the 1990s, the first aptamers and nucleic acid enzymes were successively identified through in vitro selection, an accelerated evolution process conducted in a test tube.[^36^, ^37^, ^38^] To date, in vitro selection has been extensively utilized to derive a wide variety of aptamers that specially bind to targets ranging from metal ions, small molecules, and proteins to cells.^[39]^ In parallel, relying on in vitro selection, numerous man‐made nucleic acid enzymes have been engineered with enzymatic capability for catalyzing reactions. For example, DNAzymes have been reported to catalyze RNA cleavage,[^40^, ^41^] DNA cleavage,^[42]^ RNA ligation,^[43]^ DNA ligation,^[44]^ RNA branching,[^45^, ^46^] RNA phosphorylation,^[47]^ DNA phosphorylation,^[48]^ DNA capping,^[49]^ Diels–Alder reaction,^[50]^ azide‐alkyne click cycloaddition,^[51]^ N‐acylation of DNA.^[52]^ Due to their inherent features such as chemical stability, ease of synthesis, low cost, good biocompatibility and high sensitivity, DNA aptamers and DNAzymes have been broadly applied in multiple fields, such as biosensing, disease diagnostics, therapeutics, environmental monitoring, and biomarker discovery.[^22^, ^53^, ^54^, ^55^, ^56^]

### 2.1 Selection of aptamers for pathogens

Aptamers are in vitro derived, single‐stranded DNA or RNA molecules, typically 20–80 nucleotides in length. Based on the Watson–Crick base‐pairing principle, aptamers can fold into various secondary structures, such as stems, loops, bulges, hairpins, pseudoknots, and G‐quadruplexes.^[57]^ The formation of distinct tertiary structures in aptamers is further facilitated by their secondary structures, driven by intramolecular interactions among nucleotides and intermolecular interactions between nucleotides and their target molecules. These tertiary interactions involve hydrogen bonding, van der Waals forces, hydrophobic interactions, electrostatic interactions, shape complementarity, and nucleobase π–π stacking.[^58^, ^59^] The specific tertiary conformations of aptamers enable them to recognize their cognate targets with high sensitivity and selectivity.

Aptamers present a compelling alternative to conventional antibodies, offering comparable binding affinities and specificities, coupled with high stability, smaller size, structural flexibility, scalable chemical production, and low immunogenicity. Their synthetic nature allows for precise manipulation, including the incorporation of exotic or unnatural bases and modifications to functional groups within the nucleotide structure, which enhances their functionality in biological matrices and in vivo environments. Common modifications include adjustments to the phosphodiester backbone (e.g., methylphosphonate,^[60]^ alkyl phosphomonoester,^[61]^ phosphorothioate^[62]^ and dithiophosphate^[63]^) and the pentose sugar (e.g., 2’‐fluoro, 2’‐amino, and 2’‐O‐methyl),^[64]^ as well as 3’‐end capping with inverted thymidine[^65^, ^66^] and the substitution of natural ribonucleotides with artificial counterparts (e.g., locked nucleic acid,^[67]^ unlocked nucleic acid,[^68^, ^69^] and 2’‐deoxy‐fluoro‐D‐arabinnucleic acid^[70]^). These modifications significantly enhance the biostability of aptamers and their resistance to nuclease degradation. Enhancements in binding affinity and target specificity can also be achieved through nucleobase derivatives (e.g., 5‐(*N*‐benzylcarboxyamide)‐2‐deoxyuridine)^[71]^ and 5’‐end PEGylation.^[72]^ Aptamers possess the ability to discriminate between closely related or highly similar targets, such as small molecules with different functional groups,^[73]^ homologues,^[74]^ conformational isomers,^[75]^ or even proteins with a single amino acid mutation.^[76]^ These unique features make aptamers particularly advantageous for applications in biosensing, food safety, environmental monitoring, early disease diagnostics, and targeted drug delivery.

**Figure 1:**
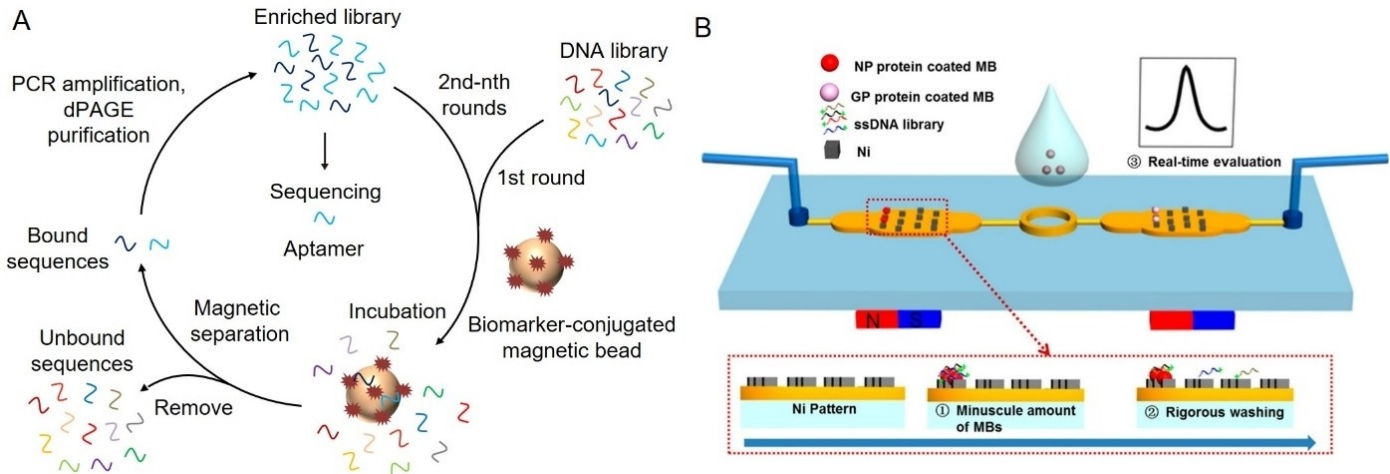
Representative methods for aptamer selection targeting pathogen biomarkers. (A) Schematic illustration of magnetic bead‐based selection of aptamers for specific biomarkers. (B) Schematic illustration of identification of aptamers against the Ebola virus using a magnetism‐controlled selection microfluidic chip. Adapted with permission from Ref.^[107]^

The aptamer selection technique SELEX was first introduced by Tuerk and Gold in 1990.^[37]^ The SELEX process begins with the incubation of target molecules with an initial oligonucleotide library, comprising approximately 10^12^ to 10^16^ sequences (Figure 1A).[^39^, ^58^] This library includes two short constant regions of 18–30 nucleotides that serve as primer binding sites, flanking a random central domain of 30–50 nucleotides. After incubation, the sequences that bind to the target molecules are isolated and amplified for the next round of selection. The selection cycle is typically repeated for several rounds, during which the DNA library becomes increasingly enriched with sequences that bind to the target, until the binding affinity of the library begins to plateau. Once this plateau is reached, the DNA library from the final selection round undergoes sequencing analysis. The resulting candidate sequences are then tested for their binding affinity to the target. Those sequences that demonstrate strong binding affinity are identified as aptamers.

#### 2.1.1 Selection of aptamers for specific biomarkers from pathogens

Aptamer selection strategies for pathogens can be divided into two main approaches: targeting specific biomarkers or whole organisms. In the biomarker‐based approach, well‐defined molecules like toxins,[^77^, ^78^, ^79^, ^80^, ^81^] structural proteins,[^82^, ^83^, ^84^] and enzymes[^26^, ^74^, ^85^, ^86^, ^87^] are selected as targets for aptamer selection (Table 1). These biomarkers are typically immobilized on solid materials, such as magnetic beads,[^79^, ^88^] membranes,[^89^, ^90^, ^91^] resins,[^92^, ^93^] or microtiter plates,[^82^, ^83^] for efficient separation of bound aptamers. The attachment is usually done via amidation reactions,[^79^, ^80^, ^84^, ^88^] allowing for strict elution of non‐specific sequences. However, immobilizing targets can block important recognition sites, and this method is not ideal for targets lacking functional groups. Additionally, biomarkers can also be immobilized on solid materials through non‐covalent methods, such as non‐specific adsorption or affinity binding. Non‐specific adsorption relies on interactions like hydrophobic forces between proteins and surfaces like microplates[^82^, ^83^] or nitrocellulose membranes,[^89^, ^90^] but it is limited to protein targets and doesn't work well for small molecules. Affinity binding, on the other hand, uses specific interactions, like biotinylated targets binding to avidin‐coated beads^[86]^ or His‐tagged proteins binding to metal ion beads (e.g., nickel or cobalt)[^74^, ^85^, ^94^] or resins,^[95]^ and maltose‐binding protein‐tagged proteins binding to amylose resins.^[92]^ To avoid blocking binding sites, Choi et al.^[96]^ and Ahn et al.^[97]^ introduced nickel beads after incubating nucleic acid libraries with His‐tagged proteins. Additionally, Li et al. used native polyacrylamide gel electrophoresis to separate DNA‐protein complexes without immobilizing the target, allowing better access to binding sites and improving aptamer selection.^[98]^

**Table 1:** List of aptamers targeting specific biomarkers from pathogens. RNA aptamers are underlined.

	Pathogens	Targets	Aptamers	Aptamer length (nt)	Sequence truncation	K_d_ (nM)	SELEX methods
**Bacteria**	*S. typhimurium* ^[89]^	OMPs	Aptamer 33	97	No	NR	Lateral flow chromatography
	*S. typhimurium* ^[108]^	LPS	EA10	80	No	28	Capture
	*B. anthracis* ^[77]^	PA toxin	Apt11	75	No	112	CE
	*E. coli* K88^[82]^	Fimbriae	aptamer 37	60	Yes	25	Microplate
	*M. aeruginosa* ^[88]^	MC–LR	RC6	96	No	61	Agarose bead
	*M. tuberculosis* ^[83]^	CFP10	CE24	88	No	375	Microplate
	*S. aureus* ^[78]^	SEA	C7	82	No	7.4	Staggered target
	*S. aureus* ^[79]^	SEC1	C10	80	No	65.14	Magnetic bead
	*C. difficile* ^[85]^	GDH	anti‐G1	82	No	3.1	Magnetic bead
	*C. difficile* ^[80]^	TcdB	R12.69	80	No	47.3	Magnetic bead
	*C. difficile* ^[74]^	RNase H2	ARH1t6	40	Yes	1.8	Magnetic bead
	*C. difficile* ^[81]^	TcdB	4937–55	NR	No	0.76	Agarose bead
		CdtA	4758–6	NR	No	0.22	
	Cyanobacteria^[109]^	CYN	CYN9	58	Yes	88.78	Agarose bead
**Viruses**	HCV^[86]^	RdRp	Aptamer B.2	92	No	1.5	Agarose bead
	HCV^[94]^	Core protein	C7	80	No	NR	Agarose bead
	HBV^[84]^	HBsAg	Aptamer H01	80	No	NR	Magnetic bead
	AI virus H5N1^[95]^	HA1	A10	79	No	NR	Affinity chromatography
	AI virus H9 N2^[96]^	H9 peptide	C7‐35 M	35	Yes	NR	Agarose bead
	SI virus H1 N1^[110]^	mini‐HA	V46	40	Yes	19.2	Magnetic bead
	SARS‐CoV^[97]^	N Protein	Aptamer 1	83	No	0.81	Agarose bead
	SARS‐CoV^[111]^	N Protein	Aptamer 1	88	No	4.93	Agarose bead
	HIV^[112]^	RT	PF1	30	No	80	Primer‐free
	GNNV^[92]^	CP	A10	95	No	4	Amylose resin
	DENV^[87]^	MTase	Aptamer #3	45	Yes	28	Agarose bead
	EBOV^[107]^	GP	GP−D01	100	No	4.1	Magnetism‐controlled chip
	CPV‐2^[90]^	VP2 protein	6 A	96	No	105.1	NC membrane
	PEDv^[113]^	N Protein	PEA1‐3	54	Yes	2.8	Magnetic bead
	SARS‐CoV‐2^[93]^	S protein	S14	44	Yes	21.8	Cobalt resin
	SARS‐CoV‐2^[98]^	S1 subunit	MSA1‐T3	37	Yes	3.1	Magnetic bead & Native gel
	SARS‐CoV‐2^[102]^	S1 subunit	nCoV−S1‐Apt1	80	No	0.33	CE
	SARS‐CoV‐2^[91]^	RBD	Apt1	51	No	290	Membrane filtration

Abbreviations: *Microcystis aeruginosa* (*M. aeruginosa)*, outer membrane proteins (OMPs), microcystin‐LR (MC–LR), 10‐kDa culture filtrate protein (CFP10), 6‐kDa early secreted antigenic target (ESAT6), *Staphylococcal* enterotoxin type A (SEA), *S. aureus* enterotoxin C1 (SEC1), penicillin binding proteins (PB2a), glutamate dehydrogenase (GDH),  toxin B (TcdB), binary Toxin A chain (CdtA), hepatitis C virus (HCV), hepatitis B virus (HBV), Avian Influenza (AI), Swine Influenza (SI), human immunodeficiency virus (HIV), Grouper Nervous Necrosis Virus (GNNV), dengue virus (DENV), Ebola virus (EBOV), Canine Parvovirus‐2 (CPV‐2), Porcine Epidemic Diarrhea Virus (PEDv), RNA‐dependent RNA polymerase (RdRp), hepatitis B surface antigen (HBsAg), hemagglutinin (HA), nucleocapsid protein (N protein), reverse transcriptase (RT), coat protein (CP), Methyltransferase (MTase). NR: Not reported.

While conventional SELEX has identified many high‐performance aptamers, novel methods have been introduced to improve selection, including capture‐SELEX, capillary electrophoresis (CE)‐SELEX, and microfluidic SELEX. Capture‐SELEX, unlike traditional bead‐SELEX, immobilizes the nucleic acid library on a stationary support to better preserve the target's native structure. This makes it effective for small molecules and low‐epitope targets but less so for targets with multiple binding sites, as this can complicate selection and reduce specificity.[^99^, ^100^] Since its introduction in 2004, CE‐SELEX has significantly improved the efficiency of aptamer selection by screening based on mobility shifts between target‐aptamer complexes and unbound sequences under a high‐voltage electric field.^[101]^ Unlike traditional SELEX, CE‐SELEX doesn't require target immobilization, allowing natural interactions. Yang et al. used this method to identify aptamers against SARS‐CoV‐2 with nanomolar affinity in just three rounds, far fewer than the typical 8–12 cycles.^[102]^ The resulting aptamer, nCoVS1‐Apt1, had a dissociation constant (K_d_) of 0.33 nM, making it the best‐performing aptamer for SARS‐CoV‐2.^[103]^ Microfluidic techniques are gaining attention for their high‐throughput and automated selection capabilities.^[104]^ These methods use microscale volumes, reducing reagent consumption and improving diffusion and separation efficiency due to short distances and large surface‐to‐volume ratios.^[105]^ Microfluidic SELEX can be adapted for personalized devices, allowing automated control and simultaneous selection of multiple targets.^[106]^ Hong et al. improved this method by combining target‐coated magnetic beads with a patterned microfluidic chip, achieving efficient washing and separation while allowing real‐time library assessment (Figure 1B).^[107]^ They successfully identified high‐affinity aptamers for two Ebola virus biomarkers (glycoprotein and nucleoprotein) in just three rounds.

Currently, most aptamer identification focuses on specific biomarkers, like the protective antigen toxin (PA toxin), from *Bacillus anthracis* (*B. anthracis*)^[77]^ and K88 fimbriae from *E. coli*.^[82]^ However, not all pathogens have unique biomarkers, making their discovery challenging. Traditional and modified SELEX methods can target common molecules, such as lipopolysaccharide (LPS)^[108]^ and RNase H2,^[74]^ by using stringent selection strategies. Even if targets are similar across organisms, aptamers with cross‐reactivity can be minimized through careful selection. For example, the DNA aptamer ARH1t6 has a K_d_ value of 1.8 nM for *C. difficile* RNase H2, significantly lower than its affinity for RNase H2 from *Bacillus subtilis* (*B. subtilis*), *E. coli*, *L. monocytogenes*, and *S. typhimurium*.^[74]^ This demonstrates SELEX's ability to isolate highly specific aptamers, even for targets not unique to a single pathogen.

#### 2.1.2 Selection of aptamers for intact pathogens

SELEX using intact pathogens as targets eliminates the need for biomarker verification and target immobilization. Any biomolecule on the pathogen's surface can potentially bind to an aptamer. This approach preserves the native conformation of these molecules, providing a more authentic environment for aptamer interactions. As a result, selected aptamers are more likely to work effectively in real samples, simplifying downstream applications like biosensing.

**Figure 2:**
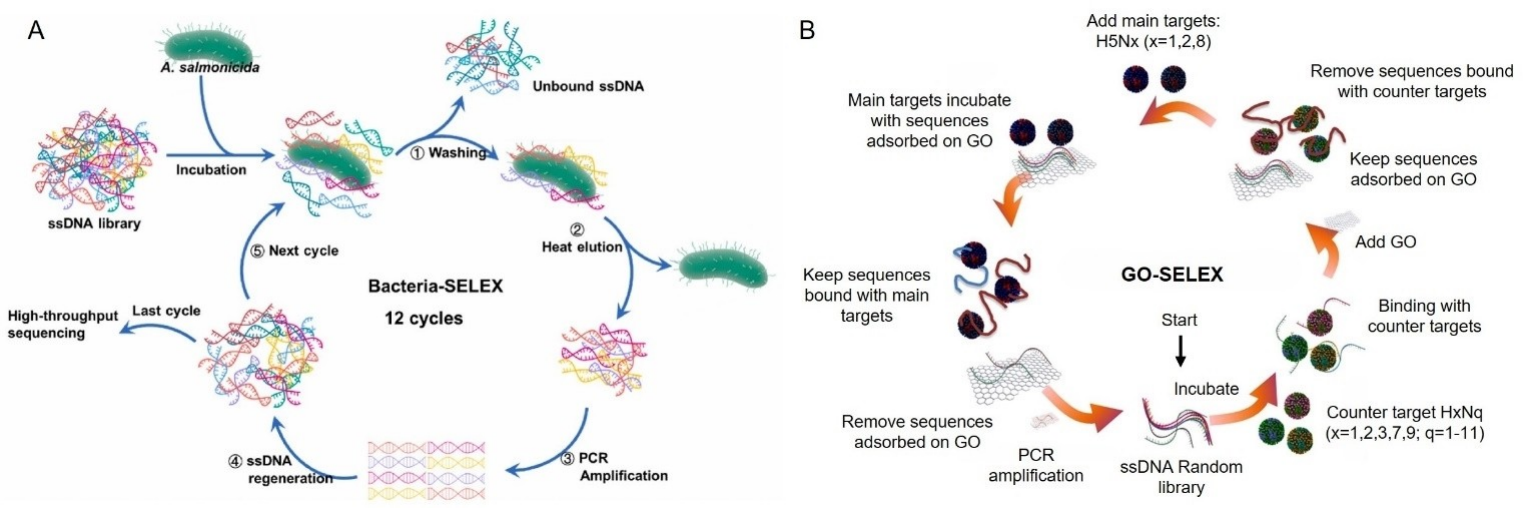
Representative methods for aptamer selection targeting pathogen cells. (A) Schematic illustration of Cell‐SELEX for selecting specific aptamers for *A. salmonicida*. Adapted with permission from Ref.^[114]^ (B) Schematic illustration of GO‐SELEX for the selection of avian influenza virus aptamer. Adapted with permission from Ref.^[115]^

Cell‐SELEX involves using live cells as targets to derive aptamers from a nucleic acid pool. This method has proven effective for screening aptamers against living organisms (Table 2), including bacteria, fungi, and cancer cells, largely due to the ease of amplifying cells through culture and separating bound from unbound aptamers by centrifugation.[^116^, ^117^] In the context of deriving aptamers for live bacteria, cells in the exponential phase are typically chosen as selection targets because they are metabolically active, with their components being highly expressed during this phase.[^118^, ^119^, ^120^] The whole‐cell selection strategy is notable for its simplicity and accessibility in aptamer development. Centrifugation efficiently isolates bacterial cell‐aptamer complexes from unbound or non‐specific sequences, avoiding the need for immobilization. This approach has been effectively used to select aptamers for *Aeromonas salmonicida* (*A. salmonicida*, Figure 2A)^[114]^ and other pathogens, including *S. aureus*,[^121^, ^122^] *Campylobacter jejuni* (*C. jejuni*),^[123]^ *E. coli*,^[124]^ *S. typhimurium*,^[125]^ *Shigella dysenteriae* (*S. dysenteriae*).^[126]^

**Table 2:** List of aptamers for intact pathogens. Note that RNA aptamers are underlined.

	Pathogens	Aptamers	Length (nt)	Sequence truncation	K_d_	SELEX methods
**Bacteria**	*S. aureus* ^[118]^	SA17	62	No	35 nM	Cell
	*S. aureus* ^[121]^	RAB35	76	No	34 ± 5 nM	Cell
	*S. aureus* ^[122]^	SA81	76	No	14.5 ± 8.2 nM	Cell
	*P. aeruginosa* ^[119]^	F23	96	No	17.3 ± 5.0 nM	Cell
	*C. jejuni* ^[123]^	ONS‐23	80	No	292.8 ± 53.1 nM	Cell
	*E. coli* O157:H7^[138]^	aptamer I‐1 two arm	64	Yes	110 nM	Cell
	*E. coli* O157:H7^[139]^	Apt‐5	80	No	9.0±2.1 nM	Cell
	*E. coli* KCTC 2571^[120]^	E1	88	No	12.4 nM	Cell
	*E. coli* NSM59^[124]^	EcA5‐27	24	Yes	110 nM	Cell
	*E. coli* DH5a^[140]^	Ec3(31)	31	Yes	225 nM	Cell
	*S. typhimurium* ^[125]^	ST2P	87	No	6.3±0.6 nM	Cell
	*S. paratyphi* A^[141]^	Apt 22	41	No	47±3 nM	Cell
	*S*. Enteritidis^[142]^	Apt 8	80	No	43.8±5.0 nM	Cell
	*S. dysenteriae* ^[126]^	Aptamer S1	87	No	23.5 ± 2.5 nM	Cell
	*P. mirabilis* ^[143]^	PmA102	66	No	3.5 nM	NC
	*L. monocytogenes* ^[144]^	LM6‐116	81	No	74.4±52.7 nM	Cell
	*H. influenzae* ^[145]^	Clone 63	80	No	0.0285±0.0043 nM	Cell
	*S. pyogenes* ^[146]^	E‐CA20	40	Yes	7 ± 1 nM	Cell
	*S. pneumoniae* ^[147]^	Lyd‐3	80	No	662 ± 111 nM	Cell
	*C. sakazakii* ^[148]^	CS4	78	No	NR	Cell
	*V. vulnificus* ^[149]^	Vapt2	149	No	26.8±5.3 nM	Cell
	*V. alginolyticus* ^[150]^	VA2	95	No	14.3±4.3 nM	Cell
	*V. parahaemolyticus* ^[151]^	Apt‐2	60	Yes	10.3±2.5 nM	Cell
	*A. salmonicida* ^[114]^	A.s‐2	104	No	32 ± 8 nM	Cell
	*B. bifidum* ^[152]^	CCFM641‐5	80	No	11 ± 0.9 nM	Cell
**Viruses**	PRRSV type II^[133]^	LB32	76	No	2.5×10^5^ TCID_50_/mL	Magnetic bead
	SMV^[153]^	SMV‐21	81	No	101 nM	Magnetic bead
	MNV^[127]^	AG3	57	Yes	NR	Filter
	MDPV^[128]^	Apt‐10	49	Yes	467 nM	Filter
	HAdV^[129]^	HAdV‐Seq4	45	Yes	0.9±0.1 nM	Filter
	SARS‐CoV‐2^[129]^	SARS2‐AR10	45	Yes	79 nM	Filter
	SI Virus H1 N1^[130]^	A20S	35	Yes	5.6 nM	Microplate
	NDV^[131]^	Apt_NDV01	41	Yes	31 nM	Microplate
	IBV^[132]^	Apt_IBV02	81	No	58.2 nM	Microplate
	BVDV type 1^[136]^	NO .11	66	No	4.08×10^4^ TCID_50_/mL	GO
	AI Virus H5Nx^[115]^	IF4	61	Yes	3×10^4^ TCID_50_/mL	GO
	AI Virus H5N2^[137]^	J_3_APT	77	No	6.913×10^5^ EID_50_/ml	GO

Abbreviations: *Pseudomonas aeruginosa* (*P. aeruginosa*), *Salmonella paratyphi* A (*S. paratyphi* A), *Salmonella enterica* Enteritidis (*S*. Enteritidis),  *Proteus mirabilis* (*P. mirabilis*), *Haemophilus Influenzae* (*H. influenzae*), *Streptococcus pyogenes* (*S. pyogenes*), *Streptococcus pneumoniae* (*S. pneumoniae*), *Cronobacter sakazakii* (*C. sakazakii*), *Vibrio vulnificus* (*V. vulnificus*), *Vibrio alginolyticus* (*V. alginolyticus*), *Bifidobacterium bifidum* (*B. bifidum*), snow mountain virus (SMV), murine norovirus (MNV), Muscovy duck parvovirus (MDPV), Newcastle disease virus (NDV), infectious bronchitis virus (IBV), human adenovirus (HAdV). NR: Not reported. NC: Nitrocellulose. GO: graphene oxide.

Whole‐virus SELEX typically utilizes either intact viruses or pseudoviruses as targets to generate specific antiviral aptamers. Unlike bacterial cells, which have micrometer‐scale diameters and can be easily pelleted via centrifugation, viruses are much smaller, with diameters in the nanometer range, requiring the use of a carrier during the SELEX separation step. Significant progress has been made in developing novel isolation strategies to efficiently select aptamers that target whole viruses, including methods based on centrifugal or membrane filters, microplates, magnetic beads, and graphene oxide. A straightforward method for isolating aptamer‐virus complexes from unbound sequences involves centrifugal or membrane filter‐based separation. This technique utilizes the molecular weight cut‐off of the filter to retain the aptamer‐virus complexes on the membrane while allowing unbound sequences to pass through. Using this approach, several aptamers with high binding affinity to viral particles have been identified. Notable examples include the aptamer AG3 for murine norovirus,^[127]^ apt‐10 for Muscovy duck parvovirus,^[128]^ HAdV‐Seq4 for human adenovirus,^[129]^ and SARS2‐AR10 for SARS‐CoV‐2.^[129]^

Another straightforward separation strategy for isolating aptamer‐virus complexes, known as Microplate‐SELEX, involves coating viral particles onto microplates via non‐specific adsorption prior to SELEX. This method efficiently removes unbound sequences without the need for sophisticated instruments like centrifuges. Recovery of aptamers from aptamer‐virus complexes is typically achieved through heat elution, as demonstrated in the selection of aptamers targeting inactivated H1 N1 virus.^[130]^ Issam Hmila and his collaborators enhanced Microplate‐SELEX by incorporating a NaCl salt gradient‐based elution method to isolate enriched aptamers targeting the Newcastle disease virus^[131]^ and infectious bronchitis virus.^[132]^

Next, magnetic bead‐SELEX is a conventional selection method that offers a convenient and cost‐effective operation without the need for expensive equipment. The separation of nucleic acid sequences from magnetic bead‐target complexes and unbound oligonucleotides can be easily accomplished using a magnetic field. Lee et al. immobilized porcine reproductive and respiratory syndrome virus (PRRSV) on tosyl‐activated magnetic beads by covalently coupling the amino groups of the viral capsid protein with the p‐toluenesulphonyl groups on the surface of the magnetic beads, facilitating the magnetic separation of aptamer‐virus complexes from unbound sequences.^[133]^

Finally, graphene oxide (GO) is a biocompatible nanomaterial with oxygen functional groups, including hydroxyl, carboxyl, and epoxy groups.^[134]^ These functional groups facilitate the physical adsorption of single‐stranded DNA (ssDNA) through π‐π stacking or hydrogen bonding. This characteristic has been harnessed in the aptamer selection method known as GO‐SELEX, which offers a rapid, efficient, and immobilization‐free approach for isolating ssDNA.^[135]^ Man Bock Gu's group applied GO‐SELEX to derive aptamers against whole viruses, including aptamer No. 11 for bovine viral diarrhea virus type 1 (BVDV type 1),^[136]^ IF4 for AI H5Nx (Figure 2B),^[115]^ and J_3_APT for AI H5N2.^[137]^

### 2.2 Selection of DNAzymes for bacteria

Since the Joyce group's landmark discovery of the first DNAzyme in 1994,^[40]^ researchers have identified numerous artificial DNAzymes from random‐sequence DNA libraries through in vitro selection.^[155]^ These DNAzymes have been harnessed across a wide array of applications including biosensing, bioimaging, drug delivery, environmental monitoring, and disease diagnosis.[^156^, ^157^] In biosensing, RNA‐cleaving DNAzymes (RCDs) have shown great potential as molecular tools due to multiple advantages, such as low cost, ease of synthesis, chemical stability, high catalytic efficiency, and compatibility with various readout and amplification techniques.^[24]^ Typically, RCDs catalyze the cleavage of RNA substrates or DNA/RNA chimeric substrates at a single ribonucleotide linkage embedded in a DNA strand. This cleavage mechanism is based on the transesterification reaction between a phosphodiester linkage and a nearby 2’‐hydroxyl group.^[41]^ To date, several RCDs have been selected for their specific responsiveness to various targets, including divalent metal ions (e.g. Pb^2+^, Zn^2+^, UO^2+^, Mg^2+^, Cu^2+^, Ca^2+^),^[158]^ monovalent metal ions (e.g. Na^+^),^[159]^ small molecules (e.g. L‐histidine^[160]^ and ATP^[161]^) and bacteria (Table 3). Our laboratory has a longstanding interest in developing RNA‐cleaving fluorogenic DNAzymes (RFDs) specific for pathogenic bacteria, which are promising candidates for bacterial bioassays. The substrates for these RFDs are structurally similar to chimeric substrates used for RCDs, with the primary difference being the incorporation of a fluorophore (F) and quencher (Q) pair on deoxyribonucleotides flanking the lone ribonucleotide. The close proximity of F and Q results in minimal fluorescence due to Förster resonance energy transfer. However, in the presence of target bacteria, the DNAzymes are activated to cleave the fluorogenic substrate at the RNA site, leading to an increase in fluorescence intensity as F and Q become spatially separated. This target‐induced signaling design uniquely combines chemical catalysis with real‐time fluorescence readouts, providing RFDs with a significant advantage for simple mix‐and‐read biosensing applications in pathogen detection.^[8]^

**Table 3:** RFDs and fluorogenic DNA/RNA substrates for bacteria.

Bacteria	Selection target	DNAzyme	Selection method
***E. coli*** ^**[162]**^	CEM	RFD‐CE1	dPAGE
***C. difficile*** ^**[163]**^	CEM	RFD‐CD1	dPAGE
***C. difficile*** ^**[164]**^	CEM	RFD‐CD2	dPAGE
***K. pneumoniae*** ^**[172]**^	Cell lysates	RFD‐KP6	dPAGE
***H. pylori*** ^**[173]**^	CEM	DHp3T4	dPAGE
***V. anguillarum*** ^**[154]**^	CEM	VAE‐2	Magnetic bead
***L. pneumophila*** ^**[174]**^	CEM	LP1	dPAGE
***P. aeruginosa*** ^**[165]**^	CEM	PAE‐1	Magnetic bead
***A. hydrophilic*** ^**[166]**^	CEM	Dah1T1	Magnetic bead
***S. aureus*** ^**[175]**^	CEM‐CIM mixture	RFD‐SA6T1	dPAGE
***V. cholerae*** ^**[167]**^	CEM	DVc1	Magnetic bead
***V. alginolyticus*** ^**[168]**^	CEM	DT1	Magnetic bead
***F. nucleatum*** ^**[176]**^	CEM	RFD‐FN1	dPAGE
***B. cocovenenans*** ^**[177]**^	CEM	RFD‐BC1	dPAGE
***K. aerogenes*** ^**[169]**^	CEM	RFA13‐1	dPAGE
***S. typhimurium*** ^**[171]**^	CEM	SSR1‐T4	dPAGE

Abbreviations: *Klebsiella pneumoniae* (*K. pneumoniae*), *Helicobacter pylori* (*H. pylori*), *Legionella pneumophila* (*L. pneumophila*), *Fusobacterium nucleatum* (*F. nucleatum*), *Burkholderia pathovar cocovenenans* (*B. cocovenenans*), *Klebsiella aerogenes* (*K. aerogenes*), crude intracellular mixture (CIM).

**Figure 3:**
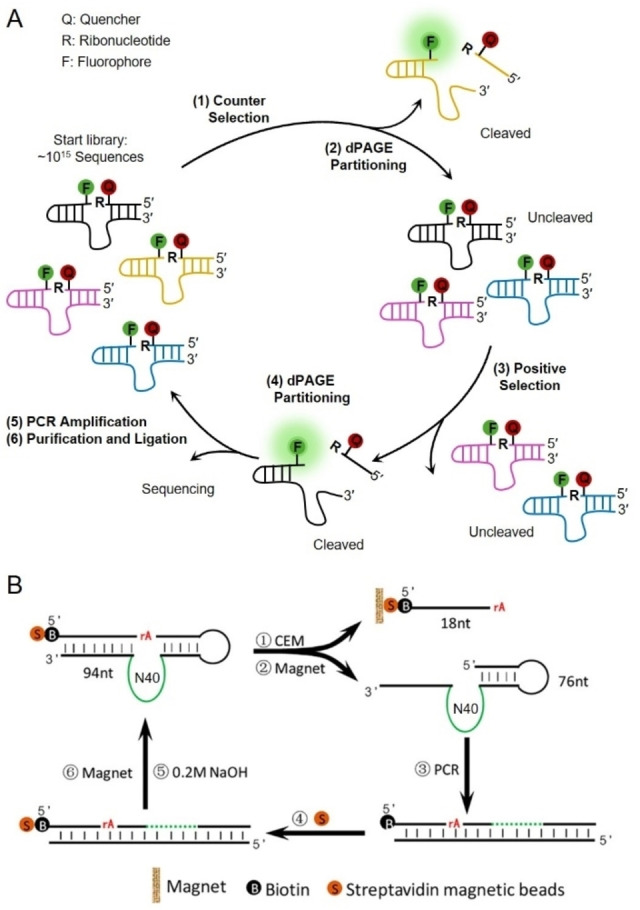
Representative selection methods of RFDs targeting bacteria. (A) Schematic illustration of a dPAGE‐based method for the selection of RNA‐cleaving fluorogenic RFDs for bacteria. (B) Schematic illustration of a magnetic bead‐based method for the selection of RFDs targeting *V. anguillarum*. Adapted with permission from Ref.^[154]^

RFDs against a target bacterium have been isolated from DNA pools with 40–70 random nucleotides. The selection target is often a crude extracellular mixture (CEM), which comes from the bacteria of interest and contains a variety of potential biomarkers like nucleic acids, proteins, lipids, and polysaccharides. This mixture is obtained by centrifuging bacterial cultures to remove the cell pellets. Using CEM allows for bypassing the need to identify a specific biomarker. Isolated RFDs hold great potential for direct bacterial detection in complex samples like serum or feces, without needing to extract or purify specific biomarkers. In a typical round of RFD selection (Figure 3A), DNA libraries are incubated with CEM from the target bacteria. Cleaved and uncleaved DNA molecules are then separated using 10 % denaturing polyacrylamide gel electrophoresis (dPAGE). Cleaved DNA sequences are collected, purified, and amplified by PCR for the next selection cycle. This approach is known as the dPAGE‐based selection method. Negative selection, which is used to remove sequences against selection buffer or CEM from non‐target bacteria, is usually performed prior to positive selection to eliminate self‐cleaving and non‐specific‐cleaving DNAzymes.

Over the past decade, several RFDs targeting bacteria have been developed using the dPAGE‐based selection method (Table 3). RFD‐EC1, the first bacteria‐responsive RFD identified by our group, was highly specific to the CEM of *E. coli*, showing minimal cross‐activity with CEM from other non‐target bacteria species.^[162]^ By integrating a cell‐culturing step, RFD‐EC1 demonstrated the capability for single‐live‐cell detection. Our group has also reported RFD‐CD1 as a strain‐specific RFD activated by BI/027‐H (Hamilton) *C. difficile*, a more virulent and antibiotic‐resistant strain.^[163]^ Its species and strain selectivity results from counter‐selection against CEM from *E. coli*, *B. subtilis*, and non‐BI/027 *C. difficile* strains used during in vitro selection. However, this high specificity limits its application in diagnosing *C. difficile* infection caused by other *C. difficile* strains. In contrast, we have also isolated RFD‐CD2, which broadly responds to *C. difficile* strains while showing no cleaving activity for other bacterial species, making it a universal DNAzyme probe for *C. difficile* infection.^[164]^ Interestingly, although positive selection was performed with CEM from the NAP1 strain of *C. difficile* and counter‐selection with a mixture of CEM from three other strains (NAP2, NAP4, and NAP7), RFD‐CD2 exhibits RNA‐cleaving activity against CEM from NAP1, NAP2, and NAP7.

In addition to dPAGE‐based selection, another widely used approach for identifying bacteria‐responsive RFDs is magnetic bead‐based selection, combined with post‐selection modifications of F and Q. For example, in the RFD selection specific for *Vibrio anguillarum* (*V. anguillarum*),^[154]^ a random DNA library was designed with a single RNA linkage as the cleavage site and a 5’ biotin group for immobilization on streptavidin‐coated magnetic beads (Figure 3B). Desired sequences were then recovered through magnetic separation. After identifying active DNAzymes (VAE‐2), the corresponding RFDs were engineered by labeling the 5’ end of the substrate strand with a fluorophore and the 3’ end of the DNAzyme strand with a quencher. Upon the complex formation of the enzyme and substrate strands after annealing, the fluorescence was quenched. In the presence of *V. anguillarum*, VAE‐2 emitted fluorescent signals due to target‐induced RNA cleavage, exhibiting high specificity and sensitivity towards *V. anguillarum*. The terminal modifications of F and Q have minimal impact on the catalytic activity and specificity of the DNAzymes. Using this method, several other bacteria RFDs have been isolated, including PAE‐1 for *P. aeruginosa*,^[165]^ Dah1T1 for *Aeromonas hydrophila* (*A. hydrophilic*),^[166]^ DVc1 for *Vibrio cholerae* (*V. cholerae*)^[167]^ and DT1 for *V. alginolyticus*.^[168]^

Recently, another class of fluorogenic DNA/RNA chimeric molecules has garnered our group's attention. These molecules serve as specific substrates for natural RNA‐cleaving protein enzymes, such as ribonucleases (RNases) from bacteria of interest, functioning as fluorogenic probes for bacterial detection. The first fluorogenic DNA/RNA molecule, RFA13‐1, was discovered by Chang et al. during the in vitro selection of RFDs for *C. difficile*.^[169]^ Upon characterization, RFA13‐1 was found to be a specific substrate for RNase I from *K. aerogenes*, distinguishing it from other RNases, including RNase A, RNase H, and RNase H2. The catalytic rate constant (*k*_cat_) of RFA13‐1 is 1.9 s^−1^, which is much higher than that of many RNA‐cleaving DNAzymes. For instance, the *k*_cat_ of the classic 10–23 DNAzyme is ~10 min^−1^.^[170]^ Additionally, we have identified a highly specific fluorogenic DNA/RNA substrate, SSR1‐T4, for RNase H2 from *S. typhimurium*. SSR1‐T4 demonstrated an EC50 (half maximal effective concentration) value of 0.048 nM for RNase H2 from *S. typhimurium*, which is 81‐ and 183‐fold lower than that for RNase H2 from *B. subtilis* and *L. monocytogenes*.^[171]^

## 3 FNA‐based colorimetric biosensors for pathogens

Colorimetric biosensors provide a simple and effective means of detecting specific analytes through visible color changes, which can be easily observed with the naked eye or measured using portable optical detectors for quantitative analysis.[^29^, ^30^] The simplicity, speed, and accessibility make this type of sensors especially appealing for point‐of‐care testing. This section will discuss pathogen detection biosensors, focusing on both non‐catalytic and catalytic colorimetric biosensors.

### 3.1 Non‐catalytic colorimetric biosensors

The rapidly advancing field of nanotechnology offers an exceptional platform for developing analytical methods, thanks to the remarkable optical, electrical, and catalytic properties of nanoparticles, which often surpass those of bulk materials.[^178^, ^179^] Gold nanoparticles (AuNPs), in particular, are highly promising as signal transducers in analytical systems, especially in colorimetric biosensors, due to their unique chemical and optical characteristics.[^180^, ^181^] The color of AuNPs is influenced by their size and shape through localized surface plasmon resonance, which gives them exceptionally high extinction coefficients. This property enables visible detection at concentrations as low as nanomolar to picomolar levels, whereas organic dyes require micromolar concentrations for similar visibility.[^181^, ^182^] Typically, AuNPs ranging from 13 to 20 nm in size, with an absorbance peak around 520 nm, are preferred in biosensor applications due to their ease of synthesis, good stability, and distinctive red color due to pronounced surface plasmon resonance.[^180^, ^183^] Additionally, the diverse shapes and biocompatibility of AuNPs facilitate straightforward surface functionalization with probes and other compounds of interest, enhancing their adaptability to various detection modalities and techniques.[^184^, ^185^]

#### 3.1.1 AuNP tag‐based colorimetric biosensors

**Figure 4:**
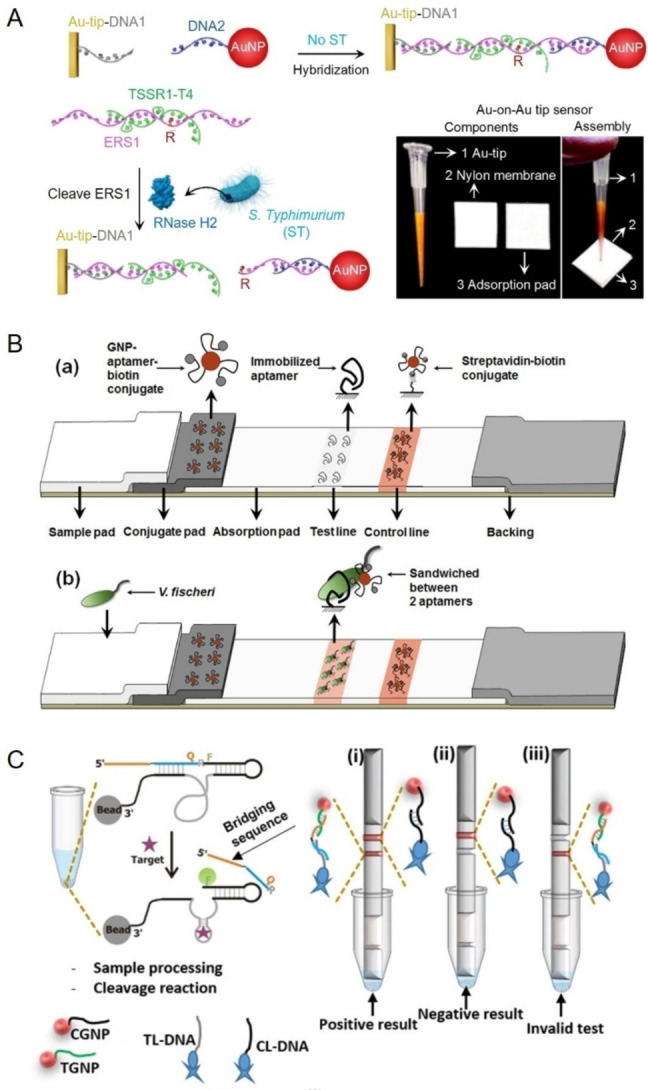
Representative colorimetric biosensors using AuNPs as tags. (A) Schematic illustration of an SSR1‐T4‐based Au‐on‐Au colorimetric biosensor for the detection of *S. typhimurium*. Adapted with permission from Ref.^[171]^ (B) Schematic illustration of an aptamer‐based lateral flow strip assay for *V. fischeri* detection. Adapted with permission from Ref.^[189]^ (C) Schematic illustration of a DNAzyme‐based lateral flow assay for colorimetric detection of *S. aureus*. Adapted with permission from Ref.^[175]^

A notable application of AuNPs in colorimetric biosensors is their use as colorimetric tags.[^181^, ^185^] In a typical biosensor, AuNPs are usually attached to FNA via Au−S bonds or biotin/streptavidin interactions. The presence of pathogens often causes the immobilization or release of AuNPs from a substrate conjugated with FNA, resulting in a change in color intensity that can be easily observed with the naked eye. Paper strips and magnetic beads are common substrates used for the construction of colorimetric biosensors. For instance, Sadsri et al. demonstrated the use of AuNPs for the construction of biosensors for *Vibrio parahaemolyticus* (*V. parahaemolyticus*) detection.^[186]^ AuNPs were first conjugated with *a*ptamers for *V. parahaemolyticus* via Au−S bonds. Magnetic beads were also functionalized with the same aptamers for target immobilization. When *V. parahaemolyticus* was present, a sandwich complex was formed among the magnetic bead, bacterium, and AuNP through specific aptamer‐bacterium recognition. The sandwich complexes were then magnetically separated, causing significant fading of the suspension color. A limit of detection (LOD) of 2.4 CFU/mL (colony‐forming unit per millilitre) for *V. parahaemolyticus* was achieved through visual inspection. In another approach, our group developed a colorimetric Au‐on‐Au tip sensor for the detection of *S. typhimurium* (Figure 4A).^[171]^ A fluorogenic DNA/RNA substrate, SSR1‐T4, for RNase H2 from *S. typhimurium* was first derived by SELEX. SSR1‐T4 was then employed to link AuNPs onto a gold‐coated pipette tip through DNA hybridization. In a typical assay, the presence of *S. typhimurium* triggered the cleavage of the SSR1‐T4, releasing the DNA‐conjugated AuNPs, which were then retained on a paper strip, resulting in a visible red dot. This portable biosensor, which requires no electrochemical or optical equipment, achieved a detection limit of 3.2×10^3^ CFU/mL for *S. typhimurium* within one hour.

In addition to magnetic bead‐based assays, paper‐based FNA biosensors using AuNPs as colorimetric tags have been developed for pathogen detection. Among these, lateral flow assays (LFAs) stand out for their rapid, sensitive, easy‐to‐use, and cost‐effective diagnostic solutions, making them especially useful in resource‐limited settings.[^187^, ^188^] A typical LFA for pathogen detection uses a sandwich format, where aptamers act as recognition elements to capture the targets and immobilize AuNPs on the test strip. This approach simplifies the test process by avoiding complex sample treatments like DNA extraction. A typical aptamer‐based LFA consists of several essential components, involving a sample pad for loading and filtering the sample, a conjugation pad containing aptamer‐functionalized AuNPs that specifically bind to the target analyte, a nitrocellulose membrane with a test line coated with aptamers to capture the target and a control line coated with complementary DNA that binds to aptamer‐functionalized AuNPs, and an absorbent pad ensures capillary flow, driving the sample and reagents through the strip. This setup has been successfully applied for the detection of various pathogens, including *Vibrio fischeri* (*V. fischeri*) (Figure 4B),^[189]^ *S. typhimurium*,^[190]^ *E. coli* O157:H7,^[190]^ *S. aureus*,^[190]^ and SARS‐CoV‐2.^[191]^

Recent innovations in enhancing AuNP and aptamer‐based LFAs for pathogens have focused on improving detection specificity and sensitivity. To improve detection specificity, Le et al. combined viral antibodies with aptamers to form aptamer‐virus‐antibody complexes on test trips.^[192]^ This approach exploits the specificity of both antibodies and aptamers avoiding false‐positive results due to non‐specific interactions, achieving over 90 % consistency in detecting influenza virus compared to quantitative real‐time PCR. To improve detection sensitivity, common approaches are integrating LFA with different nucleic acid amplification methods. In one study, Zeng's group improved detection sensitivity by integrating isothermal strand displacement amplification with LFAs. They used two aptamers targeting different outer membrane proteins: a biotin‐modified capture aptamer for bacterial immobilization and an unmodified amplification aptamer as a template for strand displacement amplification. This approach achieved an LOD of 10 CFU/mL for *S*. Enteritidis^[193]^ and 10 CFU/ml for *E. coli* O157:H7.^[194]^ In another study, Ren et al. utilized an exonuclease III‐assisted amplification strategy to enhance LFA‐based detection of *E. coli*. Their method involved the release and cyclic amplification of target single‐stranded DNA triggered by the recognition of aptamer with *E. coli*, achieving an LOD of 7.6×10^1^ CFU/mL in pure culture and 8.35×10^2^ CFU/mL in milk.^[195]^ In addition, Ying et al. employed hybridization chain reaction for highly sensitive detection of *V. parahaemolyticus* using LFA, achieving an LOD of 2.6×10^3^ cells.^[196]^ These amplification strategies significantly enhanced LFA sensitivity by 100–1000 fold compared to traditional methods without amplification.[^189^, ^190^] However, these methods typically increase testing times from 10 minutes to approximately 1 hour, posing a challenge in balancing sensitivity with rapid on‐site testing applications. Integrating FNAs with CRISPR (clustered regularly interspaced short palindromic repeats) represents a promising solution to this challenge. The highly efficient collateral cleavage activity of Cas12a (~1250 turnovers per second) on DNA strands enables rapid and robust signal amplification to deliver enhanced sensitivity in a short testing time.[^197^, ^198^, ^199^]

Bacterium‐responding RCDs can also be employed to set up AuNP‐based LFA for pathogen detection. The first such example was reported by Ali et al. for detecting *S. aureus* in nasal mucus samples (Figure 4C).^[175]^ Their approach utilized two types of streptavidin‐bound biotinylated DNA: one on the test line (TL‐DNA) and the other on the control line (CL–DNA). TL‐DNA was designed to be complementary to the 5’‐end of the cleaved fragment from an RNA‐cleaving DNAzyme. On the conjugation pad, two DNA‐gold nanoparticle conjugates were used: TGNP, which was complementary to the sequence extension of the DNAzyme specific for *S. aureus*, and CGNP, which was complementary to the CL–DNA on the control line. In the assay, the DNAzyme was first activated by *S. aureus* on magnetic beads. Following magnetic separation, the cleaved DNA fragment was transferred to the sample pad of the LFA. Driven by capillary action, this fragment bridged TGNP and TL‐DNA, resulting in a red signal on the test line. The method achieved a detection limit of 10^5^ CFU/mL when testing *S. aureus* in spiked nasal mucus samples.

#### 3.1.2 AuNP aggregation‐based colorimetric biosensors

**Figure 5:**
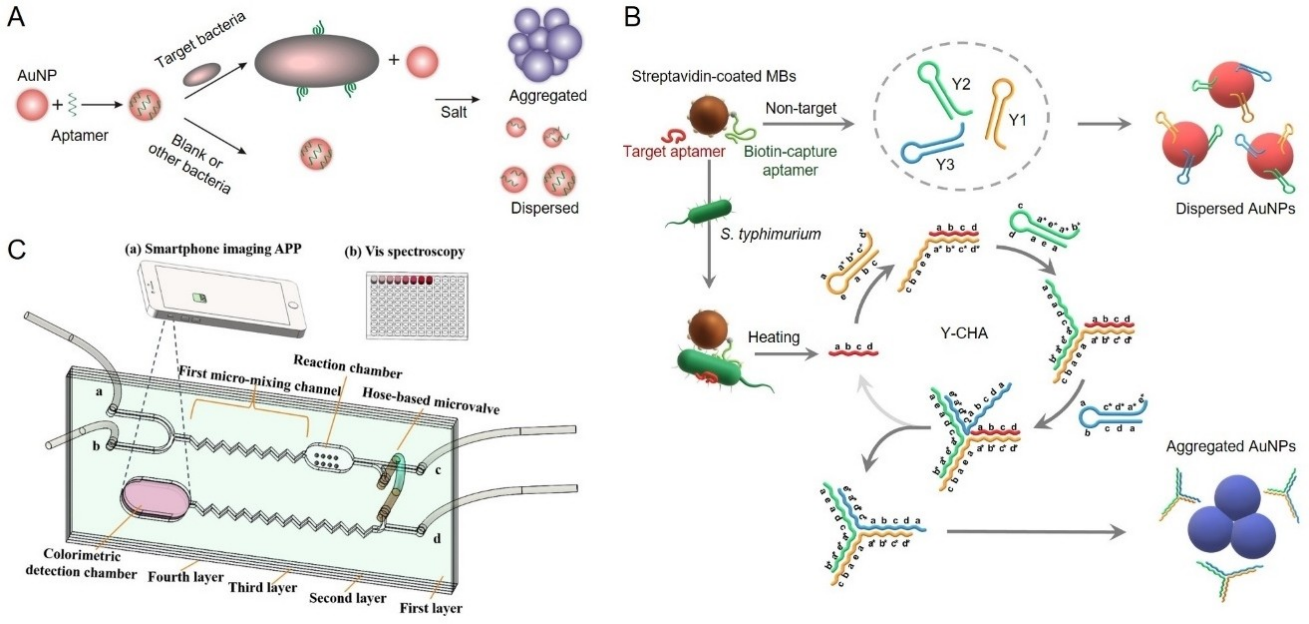
Representative AuNP aggregation‐based colorimetric biosensors. (A) Schematic illustration of a biosensor based on target‐induced aggregation of AuNPs for the detection of *E. coli* O157:H7 and *S. typhimurium*. Adapted with permission from Ref.^[201]^ (B) Schematic illustration of a target‐responsive aggregation of AuNPs integrated with an aptamer‐catalyzed hairpin assembly signal amplification approach for the detection of *S. typhimurium*. Adapted with permission from Ref.^[206]^ (C) Schematic illustration of a microfluidic chip‐based colorimetric biosensor using the crosslinking aggregation of AuNPs as a signal readout for the detection of *S. typhimurium*. Adapted with permission from Ref.^[213]^

AuNPs act as colorimetric transducers by undergoing a visible color change from red to purple as they transition from a dispersed to an aggregated state.^[200]^ Generally, AuNP aggregation‐based colorimetric biosensors include non‐crosslinking and crosslinking formats. For the non‐crosslinking format, AuNPs are initially stabilized in solution by surface‐bound capping ligands, like negatively charged citrate ions, which maintain electrostatic repulsion and keep the nanoparticles dispersed. However, the addition of salts such as sodium chloride can diminish this repulsion, promoting nanoparticle aggregation. This aggregation alters the surface plasmon resonance coupling, resulting in a shift in the absorbance spectrum and the observable color change from red to purple.^[181]^ The effectiveness of AuNP‐based colorimetric biosensors depends on controlling the dispersion and aggregation of AuNPs, which can be triggered by the binding of the MRE placed on AuNP and its target. For example, an aptamer can be attached to unmodified AuNPs and stabilize the AuNPs in high‐salt conditions via electrostatic repulsion. Upon target binding, the aptamer molecules in the conjugates detach from the AuNP surface, causing the nanoparticles to aggregate in the environment with high ionic strength and producing a visible color change from red to purple.^[180]^ The presence of targets can be easily observed with the naked eye. Wu et al. used this mechanism to develop a rapid colorimetric biosensor for detecting *E. coli* O157:H7 and *S. typhimurium* using aptamers (Figure 5A),^[201]^ achieving a detection limit of 10^5^ CFU/mL within 20 minutes, surpassing some antibody‐based sensors. The same assay principle has also been employed for the detection of *S. aureus*,^[202]^ *C. sakazakii*,^[148]^ *P. aeruginosa*,^[203]^ and *Bacillus carboniphilus* (*B. carboniphilus*),^[204]^ with demonstrated LODs of 10^6^ CFU/mL, 7.1×10^3^ CFU/mL, 10^4^ CFU/mL, 5×10^3^ CFU/mL, respectively. This AuNP‐based sensing mechanism depends on the detachment of DNA aptamers from the AuNP surface. However, the detachment of aptamers from the AuNP surface is limited due to the strong adsorption of DNA by AuNPs. For example, one study has shown that less than 5 % of the AuNP‐bound aptamers can be released upon target binding.^[205]^ Thus, a more comprehensive investigation into the broad applicability of AuNP‐based non‐crosslinking colorimetric sensors for bacterial detection is warranted.

Chen et al. improved the analytical sensitivity of AuNP aggregation‐based colorimetric sensors by incorporating a Y‐shaped catalytic hairpin assembly (Y‐CHA) amplification technique (Figure 5B).^[206]^ They developed three hairpin DNA structures (Y1, Y2, and Y3) to create a Y‐CHA circuit, which remained in a closed stem‐loop configuration due to their high melting temperatures relative to the reaction temperature. In the absence of *S. typhimurium*, the AuNPs stayed dispersed in the salt solution, exhibiting a red color because the sticky ends of the hairpins were adsorbed onto the AuNPs through electrostatic interactions. However, when *S. typhimurium* was present, a target aptamer was captured on capture aptamer‐conjugated magnetic beads through specific binding with *S. typhimurium*. The captured target aptamer was used to initiate the Y‐CHA amplification process, which resulted in the detachment of hairpin probes from the AuNP surface, causing salt‐induced aggregation of AuNPs and change of solution color from red to blue. The integration of Y‐CHA significantly increased the sensitivity of the sensing system, enabling the detection of *S. typhimurium* at concentrations as low as 2.4×10^2^ CFU/mL.

For colorimetric biosensors based on crosslinking aggregation of AuNPs, thiolated aptamers or DNA are attached to AuNPs for the preparation of nanoparticle probes.^[183]^ This approach controls AuNP aggregation through DNA hybridization or aptamer‐target binding.^[207]^ In one example, Wang et al. designed a G‐quadruplex DNA structure to regulate particle distances.^[208]^ The biosensor is composed of three main components, a G‐quadruplex DNA structure, an aptamer, and a DNA‐conjugated AuNP. In the absence of target bacteria, the aptamer binds to DNA‐conjugated AuNPs, preventing hybridization with the G‐quadruplex, and the solution remains red. When the bacteria are present, the aptamer binds to the bacteria, leading to the aggregation of DNA‐conjugated AuNPs because of hybridization with G‐quadruplex DNA structure, and the solution color changes from red to blue. This method achieved a detection limit of 1.35×10^2^ CFU/mL within 60 minutes. In another example, Gupta et al. improved sensitivity by pre‐coating AuNPs with GO to enhance aptamer loading.^[209]^ When exposed to bacteria, the aptamers bind to bacterial cell surfaces, causing the nanoparticles to aggregate and produce a visible color shift from red to blue. This method achieved a detection limit of 10^2^ CFU/mL, which was 10 times lower than the method without GO coating. In addition, Somvanshi et al. adapted the crosslinking aggregation method to a microfluidic paper‐based analytical device (μPAD) for portable and sensitive detection of pathogenic bacteria.^[210]^ μPADs, which use hydrophilic microchannels on paper separated by wax‐printed hydrophobic barriers, control fluid flow via capillary action.^[211]^ They are ideal for field‐based pathogen detection due to their quick response, low cost, and ease of use.^[212]^ Somvanshi's team designed a Y‐shaped μPAD with four zones and a central water reservoir. Aptamer‐functionalized polystyrene microsphere‐AuNPs (PS−Au‐ssDNA) were preloaded in each zone. When bacterial samples were introduced, they bound with the PS−Au‐ssDNA complexes, causing AuNP aggregation and a visible color change. The device is capable of detecting *E. coli* O157:H7 and *S. typhimurium* simultaneously, with detection limits of 10^3^ CFU/mL and 10^2^ CFU/mL, respectively. Furthermore, Man et al. developed a microfluidic chip‐based colorimetric sensor for detecting *S. typhimurium* using the crosslinking aggregation approach (Figure 5C).^[213]^ These microfluidic devices are compact portable platforms that perform various analytical processes like sample preparation, reactions, and detection.^[214]^ Their miniaturization offers benefits such as low sample volume, fast detection, and potential for point‐of‐care use.^[215]^ The sensor used a hose‐based microvalve for flow control and thiolated polystyrene (PS) microspheres to trigger AuNP aggregation through Au−S bonds.^[213]^ Bacterial samples and aptamer‐PS‐cysteamine conjugates were mixed in a micro‐mixing channel, forming complexes that can react with complementary DNA‐conjugated magnetic nanoparticles in a reaction chamber. After magnetic separation, the complexes retained in the supernatant were transferred to a colorimetric detection chamber, where cysteamine's sulfhydryl groups triggered AuNP aggregation, resulting in a visible color shift. The color change intensity, analyzed with a smartphone app and spectroscopy, was proportional to the concentration of *S. typhimurium*, with a detection limit of 6×10^1^ CFU/mL. Without *S. typhimurium*, the aptamer‐PS‐cysteamine conjugates stayed bound to the complementary DNA, preventing aggregation.

#### 3.1.3 Polydiacetylene (PDA)‐based colorimetric biosensors

PDAs represent a novel and unique class of π‐conjugated polymers, distinguished by their exceptional colorimetric and fluorescence properties. These polymers are synthesized through 1,4‐addition polymerization of diacetylene monomers, initiated by exposure to 254 nm ultraviolet (UV) or γ irradiation, which yields highly planar and uniform structures without the need for additional catalysts or initiators.^[216]^ The alternating enyne framework of PDAs enables them to absorb visible light and exhibit a characteristic blue color, due to π–π* transitions of the delocalized π electrons in the polymer backbone. Upon exposure to external stimuli such as elevated temperatures, pH changes, or specific biomolecular interactions, PDAs undergo a distinctive blue‐to‐red color transition and exhibit fluorescence activation.^[217]^ PDA vesicles, which are composed of amphiphilic diacetylene monomers with hydrophilic head groups and hydrophobic tails, are particularly effective for biosensing applications.^[218]^ The carboxylic acid termini of these vesicles can be functionalized with biomolecules, such as antibodies, enzymes, or aptamers, through amidation reaction. This functionalization enhances the ability of PDA vesicles to specifically recognize various biomolecules, making them highly valuable for biosensing applications.^[219]^

**Figure 6:**
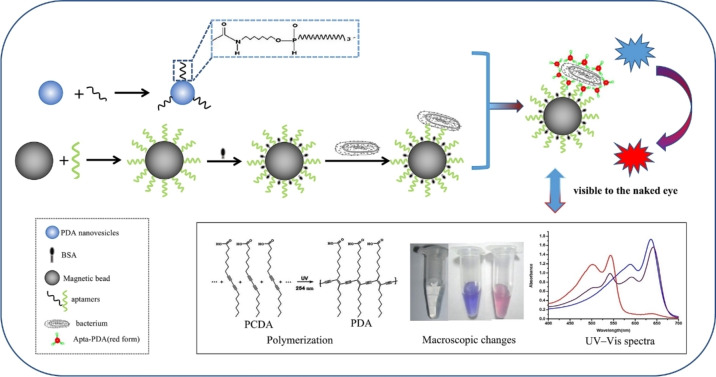
Schematic illustration of an aptamer‐functionalized PDA biosensor for the detection of foodborne pathogens. Adapted with permission from Ref.^[220]^

Wu et al. first developed a colorimetric biosensor using aptamer‐functionalized PDA vesicles for the detection of *E. coli* O157:H7.^[219]^ They utilized 10,12‐pentacosadiynoic acid (PCDA), a commonly used amphiphilic diacetylene monomer, to assemble the PDA vesicles. To facilitate aptamer conjugation, the vesicle's terminal carboxyl groups were activated using NHS and 1‐ethyl‐3‐(3‐dimethylaminopropyl) carbodiimide (EDC), creating reactive intermediates for conjugating with amino‐modified aptamers. Upon exposure to *E. coli*, these aptamer‐functionalized PDA vesicles underwent a distinct blue‐to‐red color transition, caused by the aptamer's specific binding to the bacteria, which distorted the PDA backbone or altered its polymer side‐chain arrangement. When tested on clinical fecal samples for *E. coli* O157:H7, the sensor demonstrated a high accuracy, achieving 98.5 % agreement with the standard culture method. However, the biosensor's application was limited by its detection threshold of 10^4^ CFU/mL and a reaction time of 2 hours. To overcome these limitations, Zhong et al. integrated magnetic enrichment with the aptamer‐PDA biosensor, greatly improving its sensitivity and reducing the assay time (Figure 6).^[220]^ They covalently coupled specific aptamers to magnetic beads, forming magnetic bead‐aptamer conjugates, which enabled efficient bacterial enrichment. Introducing these magnetic bead‐aptamer/bacterium complexes into the aptamer‐PDA solution created a sandwich structure (magnetic bead‐aptamer/bacterium/aptamer‐PDA), which triggered a configuration change in the PDA backbone, resulting in a color shift from blue to red. This method significantly improved the detection limit to 39 CFU/mL for *E. coli* O157:H7 and 60 CFU/mL for both *S. typhimurium* and *V. parahaemolyticus*, all within a 30‐minute reaction time. The use of aptamer‐modified magnetic beads for bacterial extraction increased detection sensitivity by 1,000‐fold while reducing the reaction time. The technique was rapid, highly sensitive, simple to perform, and required no sophisticated equipment, making it particularly suitable for resource‐limited settings.

In addition, Lee et al. developed an aptamer‐functionalized PDA liposome biosensor for the detection of *S. typhimurium*.^[221]^ The biosensor was constructed by self‐assembling PDA liposomes from a mixture of the amphiphilic diacetylene monomer 10,12‐tricosadiynoic acid (TCDA), TCDA‐NHS, and the phospholipid 1,2‐dimyristoyl‐sn‐glycero‐3‐phosphocholine (DMPC). The inclusion of DMPC improved the structural flexibility of the liposomes, which in turn enhanced the sensitivity of the sensor. Amine‐modified aptamers were conjugated to the surface of the PDA liposomes via amidation reaction with TCDA‐NHS. Upon UV irradiation, the PDA liposomes underwent polymerization, causing a color change to blue. In the presence of *S. typhimurium*, specific binding between the aptamers and the bacteria induced a color transition from blue to red, signaling detection. This aptamer‐based sensor demonstrated a detection limit of 10^4^ CFU/mL, with results obtained in just 15 minutes.

### 3.2 Catalytic colorimetric biosensors

Catalytic reactions are pivotal in enhancing the detection sensitivity of colorimetric biosensors, essential for early diagnostics, environmental monitoring, and food security. This section explores catalytic colorimetric biosensors categorized into three types: protein enzyme‐based, G‐quadruplex‐based, and nanozyme‐based biosensors.

#### 3.2.1 Protein enzyme‐based biosensors

In colorimetric immunoassays, protein enzymes such as horseradish peroxidase (HRP), alkaline phosphatase, and β‐galactosidase are widely used to improve detection sensitivity due to their exceptional catalytic properties.^[222]^ These enzymes drive oxidation or hydrolysis reactions involving specific substrates, producing a detectable color change proportional to the concentration of the target analyte. This change can either be visually observed or quantified using spectrophotometry. Key protein enzymes commonly employed for conjugation with FNAs in pathogen detection include HRP, catalase, glucose oxidase (GOx), and urease. Among these, HRP, a well‐characterized heme‐containing enzyme with over a century of study,^[223]^ is particularly favored in bioanalytical applications such as ELISA due to its small size, minimal steric hindrance, low cost, and higher turnover rate compared to other alternatives.[^224^, ^225^] HRP commonly catalyze the oxidation of chromogenic substrates such as 3,3’,5,5’‐tetramethylbenzidine (TMB), 2,2’‐azino‐bis (3‐ethylbenzothiazoline‐6‐sulfonic acid) (ABTS), and o‐phenylenediamine in the presence of hydrogen peroxide (H_2_O_2_), resulting in the formation of a colored product. With the advent of aptamers, which provide several advantages over antibodies, enzyme‐linked aptamer assays (ELAA) have emerged as an alternative to traditional ELISA. These assays, also referred to as enzyme‐linked apta‐sorbent assays, enzyme‐linked oligonucleotide assays, or aptamer‐linked immobilized sorbent assays, leverage the unique binding capabilities of aptamers.^[226]^ Similar to antibody‐based sandwich assays, aptamer‐based sandwich assays rely on the ability of targets to possess multiple binding sites.^[227]^ This allows for the formation of a sandwich complex through binding to the target.

**Figure 7:**
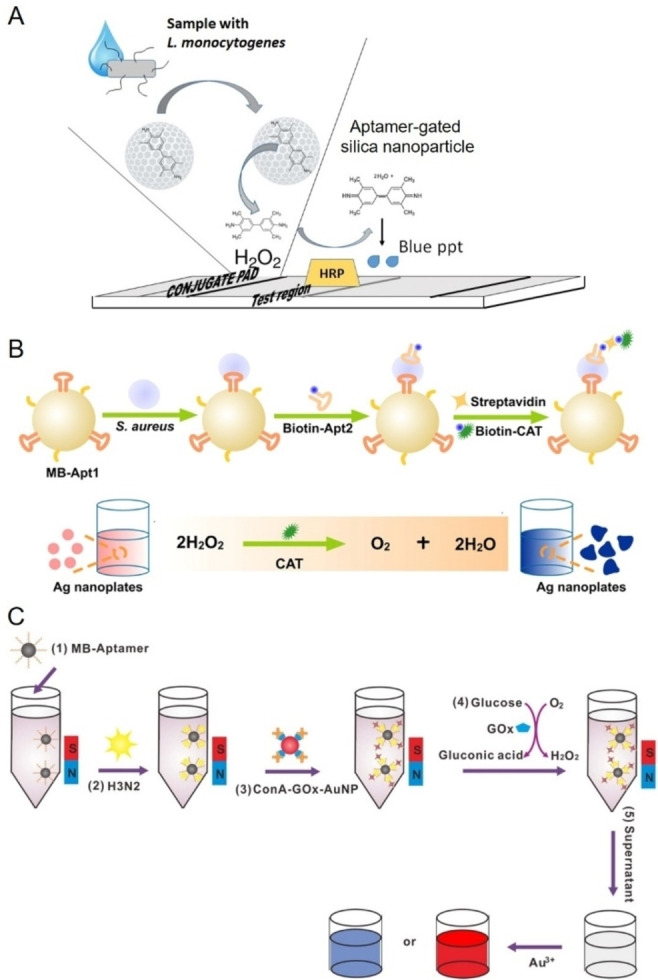
Representative aptamer and protein enzyme‐based colorimetric biosensors. (A) Schematic illustration of a lateral flow assay integrating HRP‐mediated colorimetric signal output with aptamer‐gated silica nanoparticles for *L. monocytogenes* detection. Adapted with permission from Ref.^[228]^ (B) Schematic illustration of a catalase‐based colorimetric biosensor for bacterial detection utilizing sliver nanoplates as chromogenic substrate. Adapted with permission from Ref.^[229]^ (C) Schematic illustration of a colorimetric platform for Influenza A virus detection using glucose oxidase‐conjugated AuNPs and aptamer‐functionalized magnetic beads. Adapted with permission from Ref.^[230]^

In a typical ELAA, a solid support is pre‐coated with streptavidin, followed by the immobilization of a biotin‐labeled aptamer via biotin‐streptavidin interaction. After the target is captured, an HRP‐labeled aptamer is introduced to bind to the target, which leads to substrate oxidation and produces a detectable color change.^[226]^ One commonly used substrate in HRP‐based ELAA is TMB. In the presence of H_2_O_2_, HRP catalyzes the oxidation of TMB into a blue product (TMB^+^) with absorption peaks at 370 and 653 nm. When the reaction is stopped with sulfuric or hydrochloric acid, TMB^+^ is converted into a yellow TMB diamine (TMB^2+^) with an absorption peak at 405 nm.^[231]^ This color change allows for accurate quantification of the target analyte. ELAA‐based colorimetric methods have been successfully used to detect various pathogens, including hepatitis C virus,^[232]^ Newcastle avian virus,^[131]^ Singapore grouper iridovirus,^[233]^ *S. aureus* ^[234]^ and *V. alginolyticus*,^[235]^ typically employing an aptamer‐target‐aptamer configuration. However, the bulkiness of HRP can sometimes hinder aptamer accessibility when conjugated with the enzyme. To overcome this limitation, an aptamer‐target‐antibody configuration is sometimes used, where an HRP‐conjugated antibody serves as the reporter element, as shown in the detection of Zika virus.^[236]^ Additionally, the direct‐ELAA method, where the antigen is directly coated onto the well and then incubated with an aptamer‐HRP complex, offers simplicity and has been effectively applied to the detection of human noroviruses.^[153]^ In addition, Tasbasi et al. reported a lateral flow assay for *L. monocytogenes* that integrated HRP‐mediated colorimetric signaling with aptamer‐gated silica nanoparticles to enhance sensitivity (Figure 7A).^[228]^ In their design, TMB molecules were pre‐loaded into aptamer‐gated mesoporous silica nanoparticles, which were applied to the conjugation pad. When *L. monocytogenes* cells bound to aptamers on the surface of these nanoparticles, TMB was released from the nanopores. The released TMB then migrated to the test region, where it reacted with HRP to produce a blue precipitate through an oxidation reaction with H_2_O_2_. This lateral flow assay enabled the detection of *L. monocytogenes* in minced chicken within 5 minutes, with an LOD of 53 CFU/mL.

Catalase is a tetrameric heme‐containing enzyme found in nearly all aerobic organisms, including bacteria, plants, and animals. It plays a crucial role in cellular defense by catalyzing the decomposition of H_2_O_2_ into water and oxygen, using either iron (Fe) or manganese (Mn) as cofactors. This reaction is highly efficient and does not require cellular reducing equivalents, making catalase essential for protecting cells from oxidative damage.^[237]^ Its thermal stability and high catalytic efficiency have also made it a valuable tool in bioremediation, the food industry, and increasingly, biosensing applications.[^238^, ^239^, ^240^] For example, Zhu et al. designed a catalase‐based colorimetric aptasensor for the detection of *S. typhimurium*.^[241]^ In this system, the presence of *S. typhimurium* facilitated the immobilization of avidin‐catalase on microwells through the specific interactions between aptamers and *S. typhimurium*. Upon the introduction of H_2_O_2_ and gold (III) chloride trihydrate, catalase decomposed H_2_O_2_, slowing the growth kinetics of AuNPs and causing nanoparticle aggregation, which produced a blue color in the solution. In the absence of *S. typhimurium*, AuNPs exhibited regular growth, resulting in a red color. The color difference could be visually observed or quantified via spectrometer. The method produced a detection limit of 10 CFU/mL for *S. typhimurium*. In another example, Zhao et al. reported a catalase and aptamer‐based colorimetric biosensor for detecting *S. aureus* using silver (Ag) nanoplates as chromogenic substrates (Figure 7B).^[229]^ In their system, H_2_O_2_ etches Ag nanoplates into irregular nanoplates, causing a red‐to‐blue color change. Catalase, captured on magnetic beads through specific binding of an aptamer with *S. aureus*, was used to catalyze the decomposition of H_2_O_2_, inhibiting the etching process of the Ag nanoplates. A gradual color shift from red to blue was observed proportional to the bacterial concentration. This method achieved a detection limit of 60 CFU/mL for *S. aureus*.

GOx is an oxidoreductase enzyme that plays a key role in various biological processes. It catalyzes the oxidation of β‐D‐glucose to gluconic acid and hydrogen peroxide, using molecular oxygen as an electron acceptor.^[242]^ Chen et al. developed a colorimetric detection platform for influenza A (H3N2) that leverages the catalytic activity of GOx (Figure 7C).^[230]^ In their approach, concanavalin A (ConA) was functionalized with GOx‐coated AuNPs, forming a ConA‐GOx‐AuNP complex through glycan‐ConA interactions. In the presence of the H3N2 virus, the ConA‐GOx‐AuNP complex was loaded onto aptamer‐coated magnetic beads through the specific binding of the H3N2‐specific aptamer with virus. Upon incubation with a glucose solution, GOx catalyzed the production of hydrogen peroxide. After magnetic separation, the supernatant was transferred to react with HAuCl_4_ for the generation of AuNPs. The concentration of hydrogen peroxide influenced the color and morphology of generated AuNPs. Low hydrogen peroxide levels resulted in blue, non‐aggregated AuNPs, while high hydrogen peroxide concentrations led to red, dispersed AuNPs. This approach enabled colorimetric detection of H3N2 virus at concentrations as low as 11.16 μg/mL.

**Figure 8:**
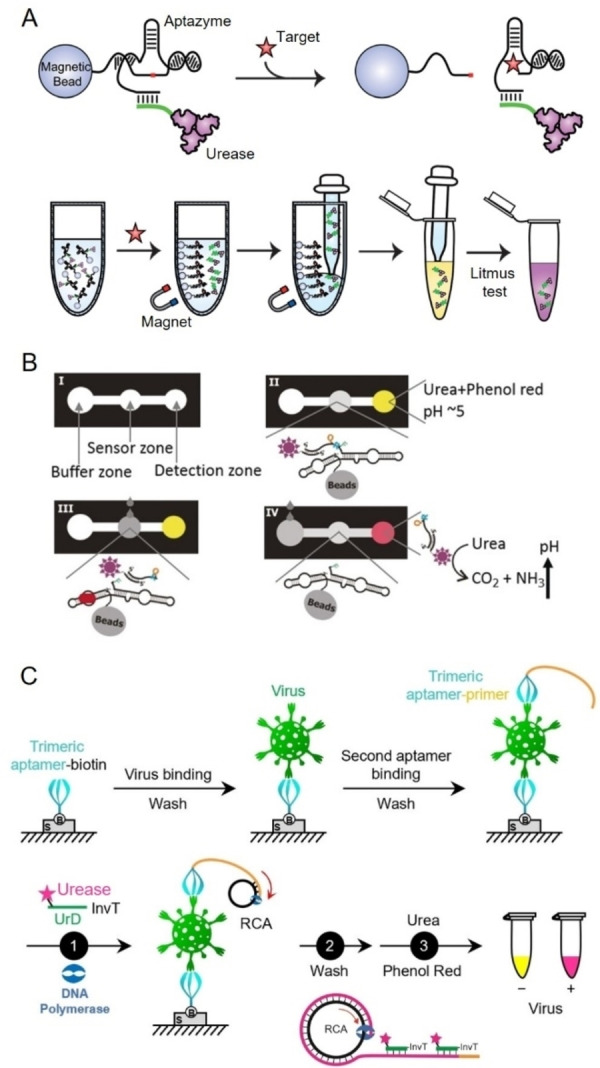
Representative urease‐based colorimetric biosensors. (A) Schematic illustration of urease‐mediated litmus test for the detection *E. coli* using an RNA‐cleaving DNAzyme as the molecular recognition element. Adapted with permission from Ref.^[245]^ (B) Schematic illustration of a urease‐based paper senor using an RNA‐cleaving DNAzyme as the recognition element for *H. pylori* detection. Adapted with permission from Ref.^[173]^ (C) Schematic illustration of a urease‐based litmus test integrated with aptamer recognition and RCA for ultrasensitive SARS‐CoV‐2 detection. Adapted with permission from Ref.^[246]^

Lastly, urease is a highly efficient enzyme that catalyzes the hydrolysis of urea into ammonia and carbon dioxide, achieving a reaction rate approximately 10^14^ times faster than the uncatalyzed process.^[243]^ Found abundantly in jack beans, soybeans, and other plant seeds, as well as in certain animal tissues and intestinal microorganisms, urease is able to modulate solution pH by catalyzing the generation of ammonia through urea hydrolysis, making it a valuable enzyme in litmus test‐based biosensing applications.^[244]^ For example, our group developed an aptazyme‐based colorimetric biosensor for *E. coli* detection using a simple litmus test for readout (Figure 8A).^[245]^ In this approach, an aptazyme for *E. coli* was employed to link urease‐DNA (UrD) conjugates on magnetic beads through DNA hybridization and biotin/streptavidin interaction. In the presence of *E. coli*, the aptazyme‐UrD complex is cleaved, releasing UrD conjugates. After magnetic separation, the released urease hydrolyzed urea leading to the generation of ammonia and increase of solution pH, which is reflected by a sharp color transition of phenol red from yellow to pink. This method achieved a visible detection of *E. coli* at 5×10^3^ CFU/mL after 1 hour incubation. We have also utilized this urease‐based litmus test to develop a DNAzyme‐based paper sensor for the detection of *H. pylori* (Figure 8B).^[173]^ The sensor was fabricated on nitrocellulose paper backed with a plastic sheet, using a wax‐printing technique, which created three spherical zones interconnected by a flow channel and surrounded by hydrophobic barriers. During the assay, bacterial samples were applied to the middle zone, which contained DNAzyme/urease/beads. This interaction triggered the cleavage of DNAzymes and the release of urease‐DNA tags. Upon the addition of running buffer to the left zone, the free urease‐DNA migrated to the right zone containing a urea/phenol red mixture. The reaction resulted in a visible color change, indicating the presence of bacteria. This method achieved an LOD of approximately 10^4^ CFU/mL for *H. pylori* in less than one hour. In addition, the reagents loaded onto the paper were stabilized using pullulan, forming a film that provided the sensor with four‐month storage stability at room temperature. Taken together, the paper sensor demonstrated several key advantages, including high sensitivity, portability, ease of use without requiring specialized expertise, and extended stability, making it a promising tool for detecting pathogenic bacteria in point‐of‐care diagnostic settings.

Additionally, we further advanced urease‐mediated biosensing by integrating rolling circle amplification (RCA) to develop ultra‐sensitive detection platforms (Figure 8C).^[246]^ This method utilized a target‐induced RCA reaction, where UrD hybridized with repeat sequences generated during RCA. The RCA products were captured by magnetic beads through the streptavidin‐biotin interaction, with unbound UrD removed via magnetic separation. The urease immobilized on the magnetic beads then catalyzed urea hydrolysis to generate ammonia, leading to the increase of solution pH and a sharp color change from yellow to pink. This approach enabled highly sensitive detection of SARS‐CoV‐2 using a universal trimeric aptamer, TMSA52, as molecular recognition elements for spike protein. Pseudovirus expressing the SARS‐CoV‐2 spike protein was successfully detected with a detection limit of 3.2×10^3^ cp/mL by the naked eye. Testing on 77 patient saliva samples demonstrated a clinical sensitivity of 83.8 % (true positive rate) and a specificity of 100 % (true negative rate). Recently, we developed an “AUT” (Aptamer‐based and Urease‐mediated Litmus Test) colorimetric assay to evaluate the diagnostic potential of monomeric, dimeric, and trimeric aptamers with real clinical samples.^[247]^ This study aimed to determine the affinity levels an aptamer must achieve for accurate clinical diagnostics. We compared two monomeric aptamers, MSA1 and MSA5, one dimeric aptamer, and two homotrimeric aptamers constructed from MSA1 and MSA5, exhibiting a 1000‐fold difference in affinity. Using the “AUT” to analyze 48 human saliva samples, we found that the trimeric aptamer assay (K_d_ ~10 pM) identified SARS‐CoV‐2 infection far more accurately than the dimeric (K_d_ ~100 pM) and monomeric (K_d_ ~10,000 pM) aptamer assays. Based on the data, we theoretically predict that the affinity levels required to achieve 80 %, 90 %, and 100 % sensitivity are 0.51 pM, 0.018 pM, and 0.00067 pM, respectively. These findings underscore the importance of developing ultra‐high affinity aptamers for highly accurate viral detection in future pandemics.

#### 3.2.2 G‐quadruplex‐based biosensors

Since Sen's group first identified the peroxidase‐mimicking activity of the G‐quadruplex/hemin complex,^[248]^ this peroxidase‐mimicking DNAzyme has gained significant attention in biosensing and has been widely explored for many bioanalytical applications.^[249]^ G‐quadruplexes are four‐stranded structures formed by guanine‐rich DNA or RNA sequences, which can adopt various topologies, including parallel, antiparallel, and mixed configurations.^[250]^ These structures are composed of planar G‐tetrads, stabilized by Hoogsteen hydrogen bonding between four guanine bases. The stability of G‐tetrads is further enhanced by the presence of cations such as K^+^, Na^+^, or NH_4_^+^, which coordinate with the oxygen atoms in the guanine bases. When two or more G‐tetrads stack through π‐π interactions, they form a stable G‐quadruplex structure.^[251]^

Hemin exhibits intrinsic catalytic activity for breaking down hydrogen peroxide. Its planar aromatic structure also enables effective interaction with the aromatic rings of guanine bases in G‐quadruplexes, allowing the G‐quadruplex/hemin complex to mimic peroxidase activity.^[252]^ Compared to traditional peroxidases like HRP, the G‐quadruplex/hemin complex offers several advantages, including sequence programmability, higher thermal stability, and lower cost. These properties have made the hemin/G‐quadruplex DNAzyme a popular catalytic label, particularly in colorimetric biosensors.[^253^, ^254^] For instance, Sun et al. developed a colorimetric aptasensor to detect *V. parahaemolyticus*, using the G‐quadruplex/hemin complex as the signal transducer and *V. parahaemolyticus* binding DNA aptamer‐conjugated magnetic beads as capture probes.^[255]^ In this system, an ssDNA containing a G‐quadruplex element and a complementary DNA element to the aptamer was initially bound to the aptamer‐conjugated magnetic beads through Watson–Crick base pairing between the complementary DNA and the aptamer. When *V. parahaemolyticus* was present, the aptamer preferentially bound to the target, causing ssDNA dissociation from the magnetic beads. After magnetic separation, the free ssDNA in the supernatant was converted into a G‐quadruplex, which, upon binding with hemin, generated a colorimetric signal when exposed to TMB and H_2_O_2_. This method achieved a detection limit of 10 CFU/mL for *V. parahaemolyticus*. Similarly, Lu et al. designed a G‐rich sequence‐modified aptamer (G‐aptamer) for the detection of *A. salmonicida*, which served both target recognition and signal transduction functions.^[114]^ They employed graphitic carbon nitride (g‐C_3_N_4_) nanosheets to adsorb the G‐aptamer through π–π interactions between the nucleobases and the tri‐s‐triazine units of g‐C_3_N_4_, preventing G‐quadruplex formation. In the presence of *A. salmonicida*, the aptamer's higher affinity for the target caused its release from the g‐C_3_N_4_ surface, allowing the G‐rich sequence to self‐assemble into a G‐quadruplex structure in the presence of K^+^. The binding of hemin to the G‐quadruplex complex then catalyzed the oxidation of o‐phenylenediamine into a yellow product in the presence of H_2_O_2_. This method achieved a visual limit of detection of 2.2×10^2^ CFU/mL for *A. salmonicida*.

**Figure 9:**
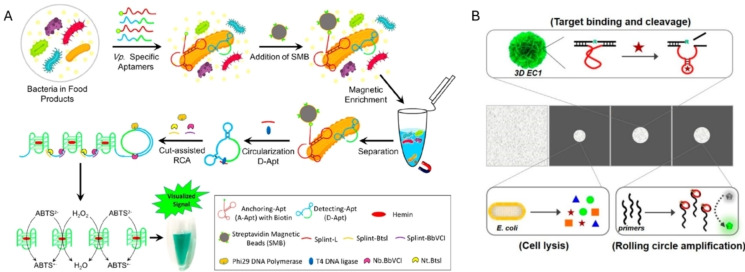
Representative G‐quadruplex‐based colorimetric biosensors. (A) Schematic illustration of G‐quadruplex‐based visualized detection of *V. parahaemolyticus* combining with cut‐assisted RCA. Adapted with permission from Ref.^[256]^ (B) Schematic illustration of an origami‐based paper sensor for rapid and portable pathogen detection using G‐quadruplex as a signaling element. Adapted with permission from Ref.^[259]^

To improve the sensitivity of G‐quadruplex‐based biosensors, various nucleic acid‐mediated signal amplification techniques have been employed. One example demonstrated by Song et al. involved the incorporation of RCA into an aptamer‐based visual sensing platform for the rapid and highly sensitive detection of *V. parahaemolyticus* in food samples (Figure 9A).^[256]^ A biotinylated anchoring aptamer (A‐Apt) attached to streptavidin‐coated magnetic beads was used to capture and concentrate *V. parahaemolyticus* cells, followed by the immobilization of a detecting aptamer (D‐Apt). D‐Apt, appended with a DNA sequence encoding a G‐quadruplex structure, served as the template for cut‐assisted RCA (CA‐RCA). This process utilized two nicking enzymes to cleave long tandem repeats generated during CA‐RCA, producing up to 10^6^ copies of monomeric G‐quadruplex sequences. Visual detection was achieved as the G‐quadruplex structures catalyzed the oxidation of ABTS^2−^ in the presence of hemin, producing a green color that is easily visible to the naked eye. This method achieved a detection limit of 10 CFU/mL for *V. parahaemolyticus* in food samples. In another study, Xu et al. designed aptamers specific to *C. sakazakii*, incorporating the antisense sequence of a G‐rich motif.^[257]^ Upon introduction of *C. sakazakii*, these aptamers were immobilized on magnetic beads through the formation of an aptamer‐bacteria‐antibody sandwich complex. After magnetic separation, the aptamers acted as templates for an exponential amplification reaction, generating amplified G‐quadruplex products that served as the peroxidase for signal readout. This approach allowed for visual detection of *C. sakazakii* within 2 hours, with an exceptionally low detection limit of 2 CFU/mL. In a third example, Chu et al. employed a cascade signal amplification strategy, where aptamer binding to target bacteria initiated self‐primer elongation for the detection of *S. aureus*.^[258]^ This process led to the production of abundant G‐quadruplex/hemin complexes, generating a highly sensitive colorimetric signal in response to the presence of *S. aureus*. The method achieved a detection limit of 3 CFU/mL for *S. aureus*, demonstrating the effectiveness of cascade amplification in bacterial detection assays. Another example was provided by Sun et al. who implemented the G‐quadruplex‐based signaling strategy within an origami‐based paper sensor for rapid and portable pathogen detection (Figure 9B).^[259]^ This design seamlessly integrated multiple critical steps, cell lysis, molecular recognition, signal amplification, and a colorimetric readout, into a compact, foldable, and user‐friendly platform. It consisted of an adsorbent pad (Panel A) and three wax‐printed reaction zones (Panels B−D). Panel B handled cell lysis and protein extraction, Panel C facilitated target‐specific DNAzyme cleavage to release a cleavage fragment, and Panel D used the cleavage fragment to carry out RCA, generating multiple units of PW17, a G‐quadruplex DNAzyme. This DNAzyme then catalyzed the oxidation of TMB in the presence of hemin and H_2_O_2_, producing a visible color change. The device was assembled using a single‐sided adhesive film that enables sequential folding, ensuring smooth transfer of reagents from one panel to the next. This integrated approach allows for the rapid detection of *E. coli* K12 with an LOD of 10^3^ CFU/mL, delivering results in just 35 minutes. The all‐in‐one design streamlines the detection workflow, making it both efficient and highly suitable for rapid, on‐site pathogen detection in resource‐limited settings.

#### 3.2.3 Nanozyme‐based biosensors

Over the past decade, various nanomaterials have garnered significant attention for their ability to mimic the activity of the naturally occurring enzyme, HRP. This includes a diverse range of metal‐based nanozymes (such as Fe, Ag, Au, Pd, Pt, and Cd), metal oxide‐based nanozymes (such as Fe_3_O_4_, CuO, and NiO), and carbon‐based nanozymes (such as graphene oxide, carbon nanotubes, and C_60_).[^260^, ^261^] These nanomaterials exhibit intrinsic peroxidase‐like properties, allowing them to catalyze the oxidation of chromogenic substrates, such as TMB, to produce a color change in the presence of H_2_O_2_.^[262]^ This peroxidase‐mimicking activity has made these nanozymes highly valuable in colorimetric biosensors. Compared to natural enzymes, they offer several advantages, including tunable catalytic activity, greater stability, lower production costs, ease of mass production, and long‐term storage capabilities.^[263]^ The use of nanozymes in FNA‐based biosensors for pathogen detection usually include two strategies, one using nanozymes as tags, the other regulating the catalytic activity of nanozymes by aptamers.

**Figure 10:**
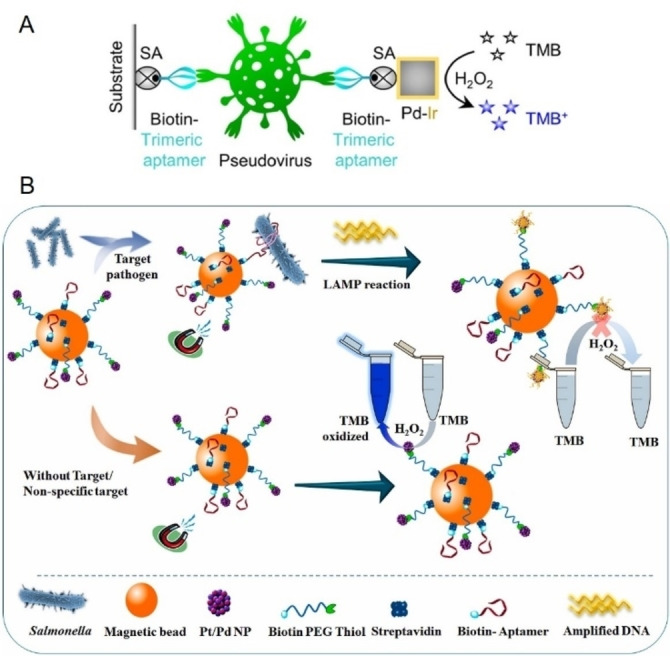
Representative nanozyme‐based colorimetric biosensors. (A) Schematic illustration of Pd−Ir nanocube and trimeric aptamer‐based colorimetric assay for SARS‐CoV‐2 detection. Adapted with permission from Ref.^[266]^ (B) Schematic illustration of colorimetric detection of *S. typhimurium* leveraging Pt/Pd‐modified magnetic beads and loop mediated isothermal amplification. Adapted with permission from Ref.^[270]^

In the approach utilizing nanozymes as tags, pathogens are typically detected through ELAA.^[262]^ In a typical nanozyme‐based ELAA, one aptamer conjugated to the nanozymes acts as the signal probe, while another aptamer, immobilized on a solid substrate such as a microplate or magnetic beads, serves as the capture probe. In the presence of target pathogen, aptamer‐conjugated nanozymes are immobilized on substrates through the specific binding of aptamers with targets. This approach has proven effective for detecting *L. monocytogenes* using Fe_3_O_4_ nanoparticle clusters as a peroxidase mimic, achieving an LOD of 5.4×10^3^ CFU/mL.^[264]^ To enhance detection sensitivity and reduce assay time, Liu et al. used aptamer‐conjugated magnetic beads to capture *L. monocytogenes* from the sample matrix, followed by the incubation with immunoglobulin Y‐coated silver nanoclusters. After washes, the immobilized silver nanoclusters were used to catalyze the oxidation of o‐phenylenediamine, resulting in a red color proportional to the concentration of *L. monocytogenes*.^[265]^ This approach enabled sensitive detection of *L. monocytogenes* at concentrations as low as 10 CFU/mL in food samples, without the need for complex sample processing procedures. In addition, we have improved the detection sensitivity of nanozyme‐based ELAA by increasing the binding affinity of aptamers.^[266]^ In our approach, a branched homotrimeric aptamer, TMSA52, was constructed using a universal aptamer MSA52 for the SARS‐CoV‐2 spike protein, a trimeric protein with three‐fold symmetry. Compared to monomeric aptamer MSA52, the binding affinity of trimeric aptamer TMSA52 increased around 2 orders of magnitude for eight differentspike protein variants. Using Pd−Ir as nanozymes, TMSA52‐based ELAA (Figure 10A) enabled sensitive detection of pseudoviruses expressing SARS‐CoV‐2 spike protein in pooled human saliva with an LOD of 6.3×10^3^ cp/mL. This method demonstrated a sensitivity of 84.0 % and a specificity of 98.3 % in analyzing 110 clinical patient saliva samples. In comparison, commercially available antibody‐based rapid antigen tests for COVID‐19 diagnosis have achieved sensitivities ranging from 48.1 % to 80 % and specificities exceeding 99.7 %, underscoring the enhanced detection sensitivity of the TMSA52‐based ELAA.[^267^, ^268^]

Moreover, nanozymes can also be employed for pathogen detection by regulating their catalytic activity through aptamers. The peroxidase activity of nanozymes is largely influenced by their surface properties, which can be adjusted through bioconjugation or the adsorption of ssDNA aptamers. Typically, the attachment of aptamers blocks the nanozyme's active sites, creating a barrier that reduces substrate accessibility. However, in the presence of the target pathogen, the aptamer undergoes a conformational change upon binding, detaching from the nanozyme surface and thereby activating the peroxidase‐mimicking activity of the nanozymes.^[269]^

Das et al. developed a colorimetric method for detecting *P. aeruginosa* by utilizing aptamer‐mediated shielding of the peroxidase‐like activity of AuNPs.^[271]^ The ssDNA aptamer, with its flexible structure, exposed nucleobases to the AuNPs, facilitating non‐specific adsorption through electrostatic attraction and π–π stacking. This interaction blocked the catalytic active sites on AuNP surface, thereby inhibiting the catalytic activity. In the presence of *P. aeruginosa*, the aptamers dissociated from the AuNP surface, restoring nanozyme activity and resulting in the oxidation of TMB, which produced a visible color change. This method demonstrated high sensitivity, detecting *P. aeruginosa* at concentrations as low as ~60 CFU/mL in water within 10 minutes. This aptamer‐mediated peroxidase inhibition strategy was also adapted for detecting *S. typhimurium* with an LOD of 7.5×10^5^ CFU/mL using magnetic nanoparticles (Fe_3_O_4_),^[272]^ and for detecting *C. jejuni* with an LOD of 100 CFU/mL using Au@Pd nanoparticles.^[273]^ To further enhance detection sensitivity, Dehghani combined LAMP with ssDNA‐based inhibition of Pt/Pd peroxidase activity for on‐site detection of *S. typhimurium* (Figure 10B).^[270]^ They first biotinylated Pt/Pd nanozymes with biotin‐PEG‐thiol, followed by sequential conjugation of aptamer‐biotin and Pt/Pd‐PEG‐biotin onto streptavidin‐coated magnetic beads, creating dual‐functional catalytic nanoparticle probes. After capturing the target bacteria, an on‐site LAMP reaction was performed to amplify the *Salmonella* hila gene. The resulting DNA products interacted with the Pt/Pd nanozymes, reducing their peroxidase‐mimicking activity. This approach achieved detection levels as low as 10–15 CFU/mL in chicken meat and 3–10 CFU/mL in whole eggs and chicken fecal samples within 3 hours. Compared to ELISA, which has a detection limit of 10^5^ CFU/mL for *Salmonella* following an 18‐hour sample processing and a 3‐hour assay, Dehghani's approach significantly improved detection sensitivity and streamlined the assay procedure.^[274]^

## 4 Conclusion and future perspectives

FNA‐based colorimetric sensors offer a powerful platform for rapid, sensitive, and specific detection of pathogens. The versatility of FNAs, particularly aptamers and DNAzymes, has been demonstrated in their ability to target specific pathogen biomarkers and intact bacterial or viral pathogens. These nucleic acids provide a reliable and flexible approach to biosensing, while the integration of colorimetric techniques, such as gold nanoparticle and enzyme‐mediated systems, enables simple and visually interpretable results. Despite these advancements, several challenges remain. The complexity of pathogen samples, issues of cross‐reactivity, and the need for robust, reproducible selection methods for FNAs demand further attention. The sensitivity of current colorimetric sensors can also be enhanced by optimizing FNA selection protocols for high affinity or catalytic activity, improving nanomaterial designs, and integrating amplification techniques. Furthermore, the translation of these technologies from laboratory settings to real‐world applications requires the development of portable, cost‐effective devices suitable for point‐of‐care diagnostics. Future perspectives in this field include the exploration of hybrid biosensing platforms that combine multiple detection mechanisms to improve sensitivity and specificity. Advances in machine learning and in silico methods for aptamer selection may streamline the discovery process, while innovations in nanomaterials and bioengineering are expected to enhance the performance of catalytic and non‐catalytic colorimetric biosensors. Furthermore, the identities of the target molecules recognized by many current FNAs selected for intact pathogens and bacterial CEMs remain undefined. Deciphering their identities represents an important step towards gaining deeper insights into the binding mechanisms of the selected FNAs, examining possible cross‐reactivities with unintended pathogens, and discovering novel biomarkers for the concerned pathogens. Additionally, current colorimetric signal output methods are primarily based on AuNPs and limited organic molecules (such as pH‐sensitive dyes and redox reagents), and are generally set up to perform single pathogen detection. Further expansion of signaling methods requires the development of more diverse colorimetric agents with distinct absorption spectra and high extinction coefficients as well as the development of powerful enzymatic approaches that can produce sharp color changes quickly at low target concentrations. These advancements can significantly enhance detection sensitivity and enable multiplex target detection. As these technologies evolve, FNA‐based colorimetric biosensors hold significant potential for widespread use in clinical diagnostics, environmental monitoring, and food safety, particularly in resource‐limited settings.

## Conflict of Interests

The authors declare no conflict of interest.

5

## Biographical Information

*Rudi Liu obtained her Bachelor's degree in Chemical Biology from Xiamen University, China, in 2012, followed by a Master's degree in Analytical Chemistry in 2015 under the mentorship of Prof. Chaoyong Yang. She is currently a PhD candidate at McMaster University in Hamilton, Canada, working under the supervision of Prof. Yingfu Li. Her research is centered on developing functional nucleic acid‐based biosensing technologies, with a particular emphasis on detecting pathogens*.

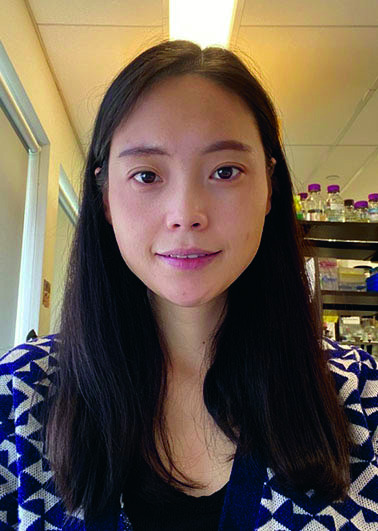

## Biographical Information

*Dr. Jiuxing Li earned his B.Sc. in chemical biology in 2011 and completed his Ph.D. in analytical chemistry in 2016 at Xiamen University under the supervision of Prof. Chaoyong Yang. Following his doctoral studies, he served as a research scientist at Michigan Technological University for a year, working with Prof. Xiaohu Xia. Currently, he is a postdoctoral fellow at McMaster University, conducting research under the guidance of Prof. Yingfu Li. His research interests include SELEX and the development of functional nucleic acid‐based diagnostics*.

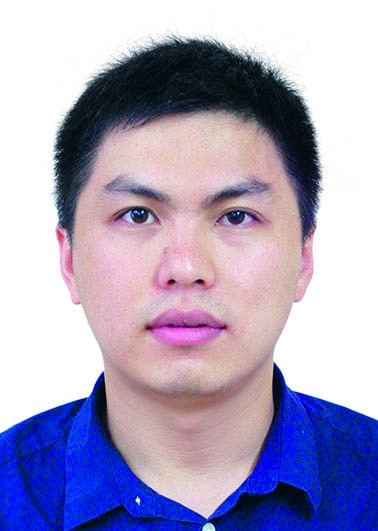

## Biographical Information

*Dr. Bruno Salena received his B. A and M.S.W from the University of Toronto in 1976 and 1978 and his M.D. from McMaster University in 1981. He completed fellowships in Internal Medicine and Gastroenterology at McMaster University from 1981–1986 and a further year completing an Advanced Endoscopy fellowship at the Cleveland Clinic. In 1988, he joined the Faculty of Medicine, Division of Gastroenterology at McMaster University where he is an associate professor. His research interests include the application of functional nucleic acids for clinical diagnosis and medical therapeutics*.

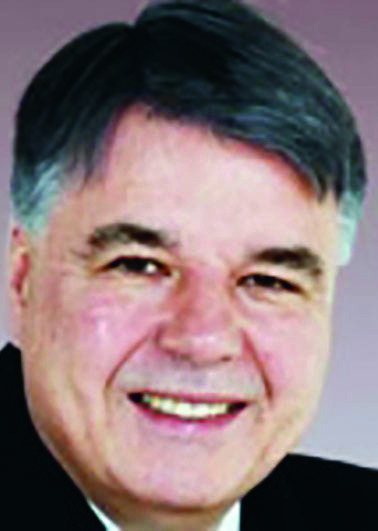

## Biographical Information

*Dr. Yingfu Li received his B.Sc. in chemistry from Anhui University in 1983, his M.Sc. in applied chemistry from China Agriculture University in 1989, and his Ph.D. in biochemistry and chemistry from Simon Fraser University in 1997 under the guidance of Prof. Dipankar Sen. He spent the next two years at Yale University as a postdoctoral fellow with Prof. Ronald Breaker. In 1999, he joined the Department of Biochemistry and Biomedical Sciences and the Department of Chemistry and Chemical Biology at McMaster University where he is now a professor. His group studies DNAzymes, aptamers, biosensors, nanotechnology, and noncoding RNA*.

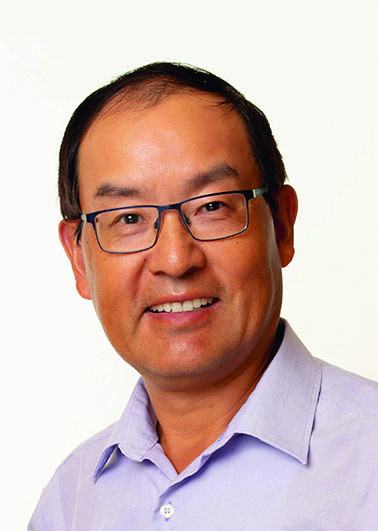

## Data Availability

Data sharing is not applicable to this article as no new data were created or analyzed in this study.
